# A Comparative Analysis of Mitogenomes in Species of the *Tapinoma nigerrimum* Complex and Other Species of the Genus *Tapinoma* (Formicidae, Dolichoderinae)

**DOI:** 10.3390/insects15120957

**Published:** 2024-12-02

**Authors:** Areli Ruiz-Mena, Pablo Mora, José M. Rico-Porras, Bernard Kaufmann, Bernhard Seifert, Teresa Palomeque, Pedro Lorite

**Affiliations:** 1 Department of Experimental Biology, Genetics Area, University of Jaén, Paraje las Lagunillas s/n, 23071 Jaen, Spain; armena@ujaen.es (A.R.-M.); pmora@ujaen.es (P.M.); jmrico@ujaen.es (J.M.R.-P.); tpalome@ujaen.es (T.P.); 2 Department of General and Applied Biology, Institute of Biosciences/IB, UNESP—São Paulo State University, Rio Claro 13506-900, SP, Brazil; 3 ENTPE, LEHNA, UMR 5023 CNRS, Université Claude Bernard Lyon 1, 69622 Villeurbanne, France; bernard.kaufmann@univ-lyon1.fr; 4 Department of Entomology, Senckenberg Museum of Natural History, 02826 Görlitz, Germany; bernhard.seifert@senckenberg.de

**Keywords:** ants, *Tapinoma nigerrimum* complex, mitogenome evolution, ant phylogeny, mitochondrial rearrangements

## Abstract

Using next-generation sequencing data, the complete mitogenomes of six species from the genus *Tapinoma* were assembled. This study explores the mitochondrial genomes of *Tapinoma* species, among them the five species from the *Tapinoma nigerrimum* complex, comparing them with each other and with other species from Dolichoderinae subfamily to understand their evolutionary relationships and evolution. *Tapinoma* mitochondrial genomes contain the typical set of 13 protein-coding genes, two ribosomal RNA genes, 22 transfer RNAs, and the A + T-rich control region. A phylogenetic analysis using the protein-coding gene sequences from available Dolichoderinae mitogenomes supports the monophyletic nature of the genus *Tapinoma*, with the *T. nigerrimum* complex forming a well-supported clade. Key findings include genetic traits unique to the *T. nigerrimum* complex, such as a start codon in the *atp8* gene and a complete stop codon in *cox1*, distinguishing them from other *Tapinoma* species. Additionally, a gene rearrangement involving *tRNA-Trp*, *tRNA-Cys*, and *tRNA-Tyr* was found exclusively in the *Tapinoma* species, suggesting a potential phylogenetic marker for the genus.

## Simple Summary

This study focuses on the *Tapinoma nigerrimum* complex, a group of five ant species, three of which have the potential to become invasive and disrupt ecosystems. Mitogenome analyses have been used to study the taxonomy, biogeography, and genetics of species. So far, only the mitogenome of one of the *T. nigerrimum* complex species has been described. In this study, we assembled and analyzed the mitogenome of the remaining *T. nigerrimum* complex species as well as two other species of the genus *Tapinoma*, which allowed a comparative study within this genus and with other species of Dolichoderinae subfamily.

## 1. Introduction

Among eusocial insects, ants, belonging to the family Formicidae (Hymenoptera), hold a remarkable place due to their higher species richness and ecological influence [1,2]. Renowned as ecosystem engineers, ants play pivotal roles in soil aeration, seed dispersal, and predation, exerting profound impacts on ecological processes. With over 14,200 described species worldwide [3], ants represent a substantial portion of terrestrial animal biomass, underscoring their ecological significance [4]. Certain ant species are recognized for their high degree of invasiveness, enabling them to proliferate globally and exacerbate the ongoing biodiversity crisis [5]. These organisms have effectively established populations outside their native habitats, resulting in significant economic and environmental repercussions in the invaded areas [6]. Species included within the genus *Tapinoma* are noteworthy in this regard. *Tapinoma melanocephalum* stands out as one of the most invasive species of ants in both hemispheres. It poses significant challenges to global biodiversity by disrupting ecosystems outside its native ranges [7]. The genus *Tapinoma*, encompassing species like *T. ibericum* or *T. magnum*, further exemplifies the invasive potential within this taxon, with implications for ecosystem health and conservation efforts [8,9].

The *Tapinoma nigerrimum* taxon actually represents a species complex. Seifert et al. [9,10] used NUMOBAT (numeric morphology-based alpha-taxonomy) to help distinguish between morphologically similar species within this group, combining morphological traits with data on nuclear DNA (nuDNA), specifically microsatellites. They identified five distinct species: *T. darioi*, *T. ibericum*, *T. magnum*, *T. hispanicum,* and *T. nigerrimum*. Similar results were achieved using microsatellite markers alone [11]. All species are native to the Mediterranean region, though the precise native ranges need further investigation, except for *T. ibericum* and *T. hispanicum*, which are only found in southern and central Spain, and *T. nigerrimum*, which is restricted to southern France and northern Spain [10]. *T. ibericum*, *T. magnum*, and *T. darioi* exhibit supercolonial behavior, with invasive potential, posing risks as potential pests [10,12]. High-resolution mapping in southern France revealed that the distribution of supercolonial and monodomous species within the *T. nigerrimum* complex (TNC) is linked to their sensitivity to urbanization [11]. The TNC is particularly significant due to its ability to control the spread of the invasive Argentine ant *Linepithema humile* [13,14]. In competition assays, the *Tapinoma* species demonstrated superior efficiency in both interference and exploitative competition, securing food within an hour and invading Argentine ant nests—behavior not observed in the latter [13]. This highlights their critical role in mitigating other invasive species’ impacts on their ecosystem [10].

Mitochondrial sequences have become valuable resources for elucidating the taxonomy, biogeography, and genetic diversity of ants, aiding in the development of strategies to mitigate their invasive spread [8,15,16,17]. Hybridization events, detectable through a mitochondrial DNA analysis when combined with morphological or nuclear DNA data, highlight the dynamic evolutionary processes shaping ant populations [9,18]. The advent of next-generation sequencing (NGS) technologies has revolutionized the acquisition of complete mitogenome sequences, enabling comprehensive investigations into ant’s evolutionary biology [12]. However, the availability of complete mitogenomes within the Dolichoderinae subfamily, particularly within the *Tapinoma* genus, and specifically the TNC, remains limited, although the mitogenome of *T. ibericum* has been recently described [16]. To address this knowledge gap, we assembled the mitogenomes of six *Tapinoma* species using NGS data, some of which are included in the TNC: *T. darioi*, *T. nigerrimum*, *T. hispanicum*, and *T. magnum*, as well as *T. madeirense* and *T. simrothi*, which do not belong to the TNC. We compared the mitogenomic characteristics of *Tapinoma* with previously sequenced Dolichoderinae mitogenomes, including other *Tapinoma* species. Gene rearrangements are common in the evolution of the mitochondrial genomes [19,20]. The comparative analysis of mitochondrial gene order is a useful tool for phylogenetic analysis because it is uncommon that the same re-arrangement occurs convergently [21]. Mitochondrial genome data have been used to analyze phylogenetic relationships at very different levels, ranging from population-level studies to phylum-level analyses [22]. By collectively analyzing these species, we aim to gain a comprehensive understanding of the genetic diversity and potential relationships within the *Tapinoma* genus, particularly focusing on the TNC, which includes several species exhibiting supercolonial behavior and the potential to become invasive [9,10]. Our approach will significantly advance knowledge of this ecologically important genus.

## 2. Materials and Methods

### 2.1. Sample Collection and Determination of Species Identity of Focal Samples

Specimens of worker ants from different species were collected in France and Spain between 2004 and 2019. Detailed information is found in Table 1. None of the species used in this study are endangered or protected, thus specific permission for their collection was not required. The workers were preserved in absolute ethanol at −20 °C until DNA extraction. The species identity of six focal samples was established using numeric morphology-based alpha-taxonomy (NUMOBAT) following the methodology described in Seifert et al. [10]. 

### 2.2. DNA Extraction, Mitogenomic Sequencing, and Assembly Strategies

About 4–5 μg of genomic DNA was isolated from a pull of 10–20 workers of each species using the NucleoSpin Tissue kit (Macherey-Nagel GmbH & Co., Düren, Germany). These DNA samples were submitted to the Novogen Company Ltd. (Cambridge, UK) for sequencing on the Illumina^®^ Hiseq™ 2000 platform (San Diego, CA, USA). A 350 bp fragment library and 151 bp paired-end sequencing reads were obtained, providing about 2.6 Gb of sequencing data for each species. To ensure high data quality, the low-quality sequences were filtered with Trimmomatic v.0.36 [23]. The mitogenomes were assembled de novo using NOVOPlasty v4.3.1 [24], which constructs organelle genomes from NGS data by extending a seed sequence. 

For the assembly of all mitogenomes in this study, the published *T. ibericum cox1* gene (GenBank accession number NC_065783) was used as the seed. This sequence was aligned with Illumina paired-end reads using bbmap (available at sourceforge.net/projects/bbmap/, accessed on 1 September 2024) and UGENE [25]. A consensus sequence was then generated and employed to initiate the assembly. Various K-mer lengths were tested, with 33 yielding the best results in terms of mitogenome completeness.

### 2.3. Mitogenome Annotation and Sequence Analysis

The mitogenomes of all the studied *Tapinoma* species were annotated following the procedure outlined by Cameron [26], using the MITOS2 web server [27,28] based on the Galaxy platform (available online: https://usegalaxy.eu/?tool_id=toolshed.g2.bx.psu.edu%2Frepos%2Fiuc%2Fmitos2%2Fmitos2%2F2.1.9%2Bgalaxy0&version=latest, accessed on 27 September 2024). The annotation of protein-coding genes (PCGs) was manually refined ensuring consistent start/stop codons and open reading frames and comparing with other Dolichoderinae mitogenomes using Geneious R11.1.5 (Biomatters Ltd., Auckland, New Zealand). Additionally, base composition estimation, the generation of circularized plots of the mitogenome, and the secondary structure analysis were conducted using Geneious R11.1.5. Codon usage analysis was performed using MEGA v.11.0.13 [29]. The resultant assemblies and annotations were deposited in GenBank under accession number (PQ459328 to PQ459333).

The final dataset of Dolichoderinae comprises 23 sequences from 18 species (Table 2). Multiple sequences of the same species were included to account for different origins and sizes. Notably, four selected mitogenomes lacked annotations and were available only as sequences (*Dolichoderus lamellosus*, *D. pustulatus*, *Leptomyrmex erythrocephalus*, and *Tapinoma sessile*). To facilitate comparisons, these mitogenomes were also annotated using the same methodology employed for the *Tapinoma* species [16].

### 2.4. Comparative Phylogenetics

Following the methodology established in the description of the *T. ibericum* mitogenome [16] for phylogenetic analysis, the complete set of PCGs from the mitogenomes of the Dolichoderinae species was used. According to the most recent ant phylogeny, published by Borowiec et al. [39], the ant subfamily most closely related to Dolichoderinae is Aneuretinae, which includes only the species *Aneuretus simoni* (Emery, 1893). Unfortunately, the mitogenome sequence for this species is not available. Therefore, we selected sequences from species of the subfamily Pseudomyrmecinae as outgroups, as it is another subfamily closely related to Dolichoderinae. These included *Tetraponera aethiops* Smith, 1877 (BK010476), and *Pseudomyrmex gracilis* (Fabricius, 1804) (BK010472). As an external outgroup, the sequence of the PCGs of *Apis mellifera mellifera* (Linnaeus, 1758) (KY926884), from Apidae family was used.

The alignment of the concatenated PCGs was performed using MAFFT v7.453 software [40]. The phylogenetic relationships were then reconstructed using the maximum likelihood (ML) method implemented in MEGA v.11.0.13 [29] employing the GTR + G + I model (model with the lowest Bayesian information criterion) with 1000 bootstrap replicates.

The genetic distances for the PCGs were estimated using the R package ape_5.4-1 [41] and graphically plotted as a heatmap using the R package ggplot2 [42]. Only a single sequence of *L. humile* (KX146468) was considered, as it is the most central sequence in the phylogenetic tree and sufficient for this purpose. There are four *D. sibiricus* mitogenome sequences deposited in GenBank. The phylogenetic analysis indicates that these sequences do not form a single cluster; instead, they appear in well-supported, distinct clades, with two sequences grouped in one clade and the other two in a separate clade. As we are uncertain whether these represent different species, we selected one sequence from each clade for further analysis of the PCGs’ genetic distances (MT919976 and MK801110).

## 3. Results and Discussion

### 3.1. General Features and Phylogenetic Analysis

**Figure 1:**
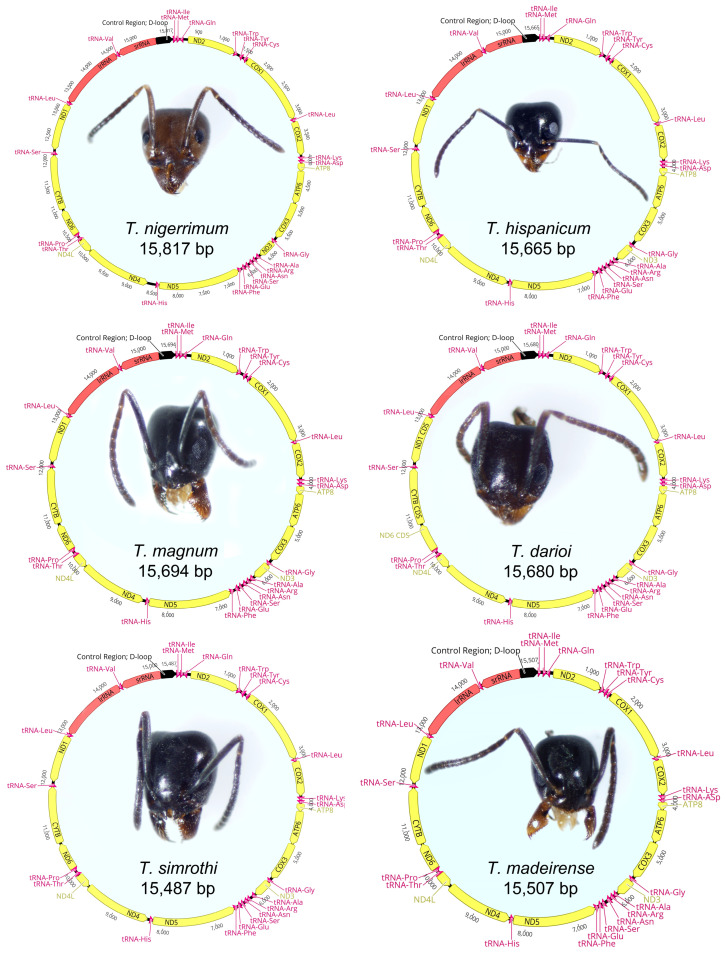
Graphical maps of the mitogenomes for four species from the *T. nigerrimum* complex (*T. nigerrimum*, *T. hispanicum*, *T. magnum*, *T. darioi*), as well as for *T. simrothi* and *T. madeirense*.

The complete mitogenomes of *T. darioi*, *T. nigerrimum*, *T. hispanicum*, *T. magnum*, *T. madeirense*, and *T. simrothi* were assembled and annotated (Figure 1, Supplementary Table S1). The new mitogenome sequences ranged in size from 15,487 bp (*T. simrothi*) to 15,817 bp (*T. nigerrimum*). These sizes are comparable to those found in previous studies for *T. ibericum* (15,715 bp), *T. melanocephalum* (15,499 bp), or *T. sessile* (15,287 bp), as well as other sequenced Dolichoderinae mitogenomes, in which the mitogenome sizes vary between 15,287 and 16,259 bp (Table 2). The determined sequences are the typical double-stranded circular molecules that, like most eukaryotic mitogenomes, encode a total of 37 genes (13 PCGs, 22 tRNAs, 2 rRNAs) and include an A + T-rich control region. 

**Figure 2:**
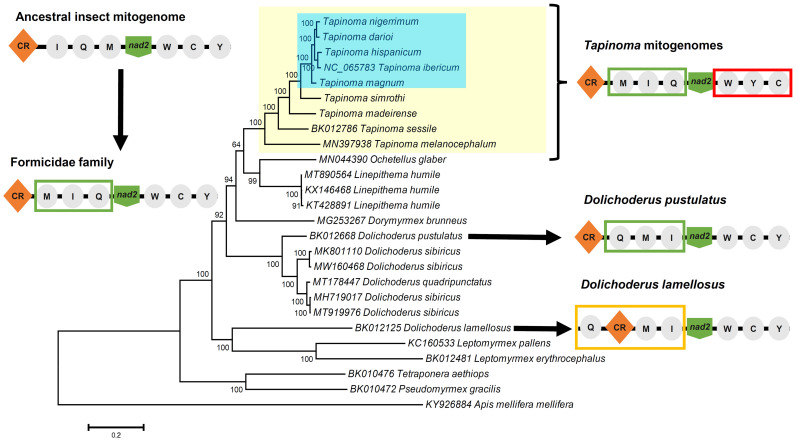
Phylogenetic tree based on Dolichoderinae mitogenomes using the ML method. Bootstrap values above 70 are shown next to the branches. Species from the *T. nigerrimum* complex are shaded in blue, while the remaining species from the *Tapinoma* genus are shaded in yellow. *T. aethiops* and *P. gracilis*, from the Pseudomyrmecinae subfamily, were also included. The tree was rooted using *A. mellifera mellifera* as an outgroup. Mitochondrial gene rearrangements found in Dolichoderinae in relation to the ancestral insect mitogenome are also depicted. tRNAs clusters which are different from the ancestral insect mitogenome are highlighted in green, red, and yellow squares.

Concatenated sequences of the PCGs were used for phylogenetic analyses. This analysis includes all sequenced mitogenomes from species of Dolichoderinae and the six mitogenomes sequenced and annotated in this study. The maximum likelihood tree (Figure 2) showed that all species from the genus *Tapinoma* are grouped into a well-supported clade with a high bootstrap value (100%), corroborating that the genus is monophyletic, as was observed in previous analyses [16,43,44]. Among the included *Tapinoma* species, *T. melanocephalum* exhibits a basal position, potentially reflecting earlier divergent evolution within the genus. 

**Figure 3:**
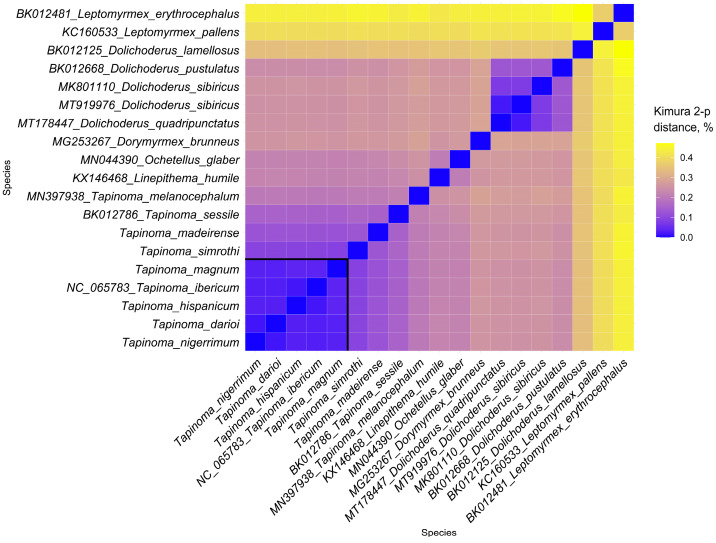
Heatmap summarizing the genetic distance values of PCGs in Dolichoderinae mitogenomes, calculated using the Kimura 2-parameter model. The genetic distances within species of the *T. nigerrimum* complex are highlighted with a black square.

Species of the TNC are grouped in a well-supported clade. This species complex includes *T. nigerrimum*, *T. darioi*, *T. hispanicum*, *T. ibericum*, and *T. magnum*. Seifert et al. [9,10] and Centanni et al. [11], through NUMOBAT and molecular analyses involving *cox1* and microsatellite markers, concluded that this species complex comprised five species. The results obtained with complete mitogenomes are strongly consistent with previous studies, identifying *T. ibericum* and *T. hispanicum* as sister species, as are *T. darioi* and *T. nigerrimum*. Additionally, *T. magnum* is confirmed as the most distantly related species, consistent with previous studies. When compared to the phylogeny in Seifert et al. [9], which did not feature *T. hispanicum*, our results are broadly similar, already showing a sibling species relationship between *T. darioi* and *T. nigerrimum*. Analysis of the genetic variability using PCG pairwise genetic distances, measured with the Kimura two-parameter model, reveals genetic distance values lower than 0.1% among all species in the TNC (Figure 3), suggesting that the species within this complex are genetically closely related, possibly due to recent evolutionary divergence. For this analysis, only one of the available sequences was used for each species, as in the case of *L. humile*, a species for which three different sequences have been deposited in GenBank (Table 2). The case of *D. sibiricus* is different. The phylogenetic analysis shows that the four mitogenome sequences of this species cluster into two different clades with high bootstrap values. We cannot determine whether there is an issue with species misidentification or whether *D. sibiricus* includes cryptic species. In any case, one sequence from each clade was selected. Species identities can be established using adequate markers of nuclear DNA or expression products of nuclear DNA that are least influenced by environmental modification [45]. The exchange of matrilines between species, mostly by ancient or current hybridization but also by incomplete lineage sorting, is a frequent event in ants [46]. This poses a risk for using mitochondrial genomes in phylogenetic studies. *Tapinoma* ants are among the ant genera in which the transfer of mtDNA between lineages defined by nuclear DNA or its expression products is comparatively rare—found in 6% of samples [9]. The low rate in *Tapinoma* is probably due to well-differentiated male genitalia or intranidal mating in the supercolonial species [9]. This reduces the frequency of interspecific hybridization. We established the identity of the focal *Tapinoma* samples (Table 2) by the use of NUMOBAT, which showed a 98.3% agreement with classification by nuclear DNA in both the *nigerrimum* and *simrothi* groups [10]. We consider the risk of transfer of matrilines between lineages defined by the nuclear genome as low in the cases presented here. We are aware that whole-genome sequencing of nuclear DNA would answer this question.

### 3.2. Gene Organization and Sequence Analysis

The comparative analysis of the mitogenomes in arthropods has enabled the identification of an ancestral mitogenome for this group of organisms [47]. The gene order of this ancestral mitogenome is also considered ancestral in insects [48]. In Formicidae, the main change with respect to the ancestral insect mitogenome affects the region located between the control region and the *nad2* gene, which includes the *tRNA-Ile*, *tRNA-Gln*, and *tRNA-Met* genes (CR-IQM-nad2) (Figure 2). In most of the ant mitogenomes analyzed, including the species of Dolichoderinae, the order of these transfer RNAs has changed to MIQ [16], which could be considered an ancestral or plesiomorphic character in ants. In addition to this change, three other rearrangements have been detected in Dolichoderinae species in relation to the ancestral mitogenome (Figure 2). The first affects *D. pustulatus*, in which a second change occurs in the same transfer RNAs, showing the order QMI. In *D. lamellosus*, a translocation of the *tRNA-Gln* gene has occurred, placing it between the *srRNA* gene and the control region. The third change appeared in species of the genus *Tapinoma*, affecting the tRNAs located downstream of the *nad2* gene, which in *Tapinoma* shows the order WYC (*tRNA-Trp*, *tRNA-Tyr*, *tRNA-Cys*), unlike other ants that present the ancestral order WCY [16]. This difference is maintained in all TNC species as well as in the remaining *Tapinoma* species. The new data support the hypothesis of Ruiz-Mena et al. [16] that this change could be considered a synapomorphic trait of the genus *Tapinoma* and that it could potentially be used as a molecular marker to establish the boundaries of the genus.

Like typical eukaryotic mitogenomes, the *Tapinoma* mitogenomes encode 13 PCGs (Figure 1, Supplementary Table S1). Most PCGs are encoded by the heavy strand (H-strand), while the *nd4*, *nd4l*, *nd5*, and *nd1* genes are located on the light strand (L-strand). All *Tapinoma* PCGs initiate with the standard ATN codon. The main difference between species of the TNC species and other *Tapinoma* species was found in the start codon for the *atp8* gene (Table 3). Outside of the TNC, this gene has ATA or ATT as the start codon. However, in four of the TNC species, the start codon is ATC. Within the TNC, only *T. magnum* has ATT as the start codon. The phylogenetic analysis shows that *T. magnum* occupies a basal position in the clade of TNC species. It is plausible to assume that the acquisition of the ATC codon is a synapomorphic trait that appeared in the common ancestor of the other four TNC species. 

In the TNC species, all PCGs present the TAA stop codon. However, incomplete stop codons were observed in non-TNC species, specifically in the *nad2*, *cox1*, and *nad5* genes (Table 3). Incomplete stop codons (TA- or T--) occur when the coding sequence ends within the 5′ end of the adjacent tRNA, with a functional stop codon generated by the addition of a poly(A) tail at the 3′ end prior to transcription [49,50]. The existence of complete stop codons in all genes in the TNC suggests a potential evolutionary shift toward a more stable and efficient genomic architecture. The main difference was found in the *cox1* gene, which presents an incomplete stop codon in all species outside the TNC. Hence, the presence of the complete stop codon for this gene seems to be a synapomorphic trait in all TNC species. 

Mitogenomes exhibit two distinct non-coding sequences: the control region (CR) and the intergenic spacers (IGSs). The size variations observed among the mitogenomes of different *Tapinoma* species were primarily attributed to differences in the IGS regions and, most notably, the CR. In the mitogenomes of *Tapinoma* species included in this study, the length of the CR ranges from 318 bp in *T. simrothi* to 356 bp in *T. madeirense*. Additionally, 27 IGSs were identified (Supplementary Table S1). These IGSs vary in length from 1 to 102 bp, with the longest intergenic spacer found between the *tRNA-Gln* and *nad2* genes in all the species, ranging from 69 to 102 bp. When comparing *Tapinoma* species to other ants for which IGSs have been described, the total number of IGSs in the *Tapinoma* species is comparable to that found in other Dolichoderinae ants [16]. In species within the TNC, the cumulative size of all IGSs ranges from 571 bp to 697 bp, consistent with the size found in *T. ibericum* (719 bp). In contrast, in species outside the TNC, the size of the IGSs ranges from 423 bp (*T. madeirense*) to 455 bp (*T. simrothi*). This is notably smaller than what is found in other ant species, such as *Solenopsis invicta* (Buren, 1972) (519 bp) and up to nearly 4 kb in *Atta laevigata* (Smith, 1858) [51,52].

Gene overlaps in the *Tapinoma* species were observed at two gene junctions. The first one was found between the *tRNA-Ile* and *tRNA-Gln* genes in all TNC species. In all TNC species, this overlap is three bp in length. *T. simrothi* also exhibits a three bp overlap, but this overlap was not found in *T. madeirense*, *T. sessile,* or *T. melanocephalum* (Supplementary Table S1). In *T. sessile* and *T. madeirense* there is an IGS between the *tRNA-Ile* and *tRNA-Gln* genes with one or two bp, respectively. However, this region in *T. melanocephalum* is 67 bp in length and contains a tandem repeat sequence (TAACTAACT). The second gene overlap, measuring seven bp (ATGATAA), occurs between the *atp8* and *atp6* genes, appears in all *Tapinoma* species and is fully conserved across the Dolichoderinae mitogenomes [16]. Indeed, the *atp8*/ *atp6* gene junction is highly conserved among arthropods [26,53], but in other hymenopteran species it was also possible to find an IGS in the *atp8*/*atp6* junction such as in the wasp *Evania appendigaster* (Linnaeus, 1758), in which the *atp6* and *atp8* genes are separated by an IGS of 244 bp [54].

**Figure 4:**
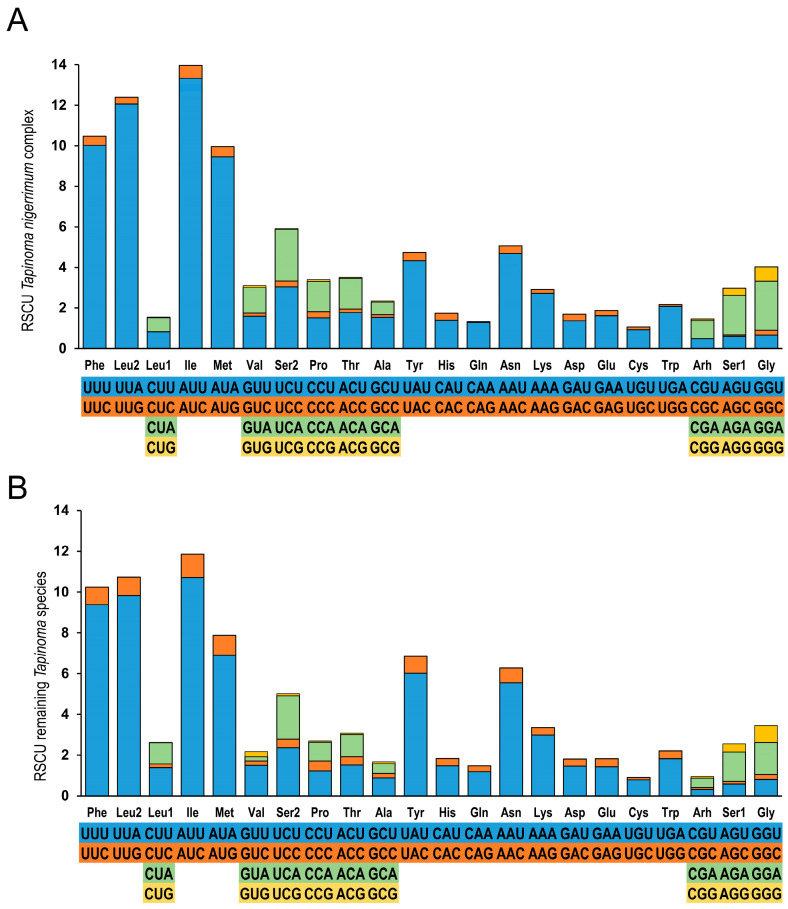
The codon usage in PCGs of *Tapinoma* mitogenomes. (**A**) RSCU in mitogenomes of species from the *T. nigerrimum* complex genus. (**B**) RSCU in mitogenomes of the remaining *Tapinoma* species.

Overall, the A + T nucleotide content in these mitogenomes is significantly higher than that of G + C and is a characteristic shared by Hymenoptera mitogenomes [55]. The A + T content in the TNC is notably high, reaching approximately 84%, and this pattern is also observed in species not included in the TNC described in this article, with A + T percentages varying within a narrow range. The lowest percentage was found in *T. madeirense*, with an A + T content of 83.6%, and the highest value was found in *T. nigerrimum*, with a percentage of 84.3%. Codon usage also reflects this bias toward A + T-rich codons (Figure 4, Supplementary Table S2). This bias in the utilization of codons for the same amino acids can be quantified through relative synonymous codon usage (RSCU) values in the mitogenome PCGs. RSCU indicates the observed frequency of a codon in a gene relative to the expected frequency under equal codon usage. For all synonymous codons, the RSCU value is higher in codons ending in A or T (NNA or NNT). The most frequently used codons are A + T-rich: ATT (Ile), TTA (Leu), TTT (Phe), and ATA (Met). Synonymous codons ending in A or U are more prevalent than those ending in G or C. For example, UUU (RSCU = 9.9) is more common than UUC (RSCU = 0.1) for Phe. The preference for codons ending in A or T appears to be a general characteristic in insects and has been observed across several insect groups [56,57,58,59].

The amino acid compositions of the *Tapinoma* PCGs are similar within the TNC as well in the remaining *Tapinoma* species, probably because of evolutionary conservation, optimization of translation efficiency, and similar selective pressures that maintain functional integrity. However, we found that their proportions are not exactly the same: Ile was observed as the most commonly represented amino acid, followed by Leu, Phe, and Met, and therefore, the codons corresponding to these common amino acids also have relatively high proportions. The A + T bias in usage can also be seen in the stop codons. In all *Tapinoma* mitogenomes, all the used stop codons are TAA (13 times), which, in some cases, is incomplete as mentioned above, while the TAG stop codon is not used at all. The TAG stop codon is also not present in the PCGs of the other *Tapinoma* species (Supplementary Table S1). Most of the PCGs described in the mitogenomes of Dolichoderinae use the TAA stop codon or its incomplete variants. The exceptions to this are the *nad3* and *nad4l* genes in *L. erythrocephalus* and the *nad1* gene in *L. humile*, in which the TAG stop codon is present.

**Figure 5:**
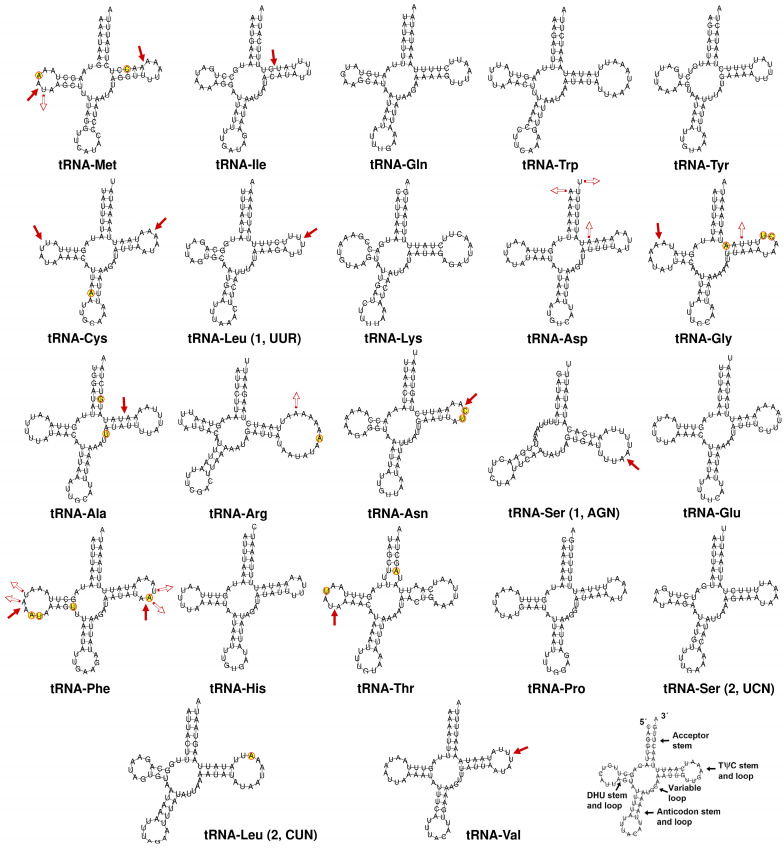
Putative secondary structures of the 22 tRNA genes in the *T. nigerrimum* complex. Sites that are fully conserved among species are indicated with black nucleotides. Red circles indicate positions in which nucleotide substitution has taken place in relation to the *T. ibericum* tRNA. Hollow arrows indicate sites with deletions, while solid arrows indicate sites with insertions.

All the mitogenomes described in this article exhibit the typical 22 tRNAs, as is usual in insects [20], although the existence of additional tRNAs has been described in some Poneroid ants, likely originated from duplications [60]. In the TNC, the size of the tRNAs range from 58 bp (*tRNA-Ser1* gene in *T. madeirense*) to 75 bp in the *tRNA-Arg* gene of *T. ibericum* (Supplementary Figure S1). These values are similar to those reported in other Dolichoderinae species [32]. Figure 5 displays the 22 tRNAs found in *T. ibericum* [16] and the changes found among TNC species. All tRNAs can fold into the typical secondary structure, except for tRNA-Ser1 (AGN), which lacks the stable sequence in the DHU arm, which is a common feature among insects and other metazoans [61,62,63]. The sequence of 8 out of the 22 tRNAs is identical across all species within the TNC (Figure 5). However, changes can be observed when comparing the sequences of these eight tRNAs to those found in other *Tapinoma* species that are not part of the TNC (Supplementary Figure S1). Among TNC species, *T. magnum* exhibits the greatest variability compared to the other four species, likely due to its more distant phylogenetic relationship. The variable positions in the tRNAs are mainly found in the DHU and TΨC arms, likely because they are less constrained by structural requirements, allowing for evolutionary flexibility. The anticodon arm is identical in 21 of the 22 tRNAs across the TNC species (Figure 5). This arm contains the anticodon triplet, which is crucial for pairing with complementary mRNA codons in the ribosome. Mutations in this region could have severe effects, as they might directly impact the tRNA’s ability to recognize its corresponding codon. The only change observed in the anticodon arm occurs in the tRNA-Cys of *T. hispanicum*, in which an adenine is replaced by a guanine. This change results in an A-U to G-U pairing in the stem of this arm, which theoretically would not affect the secondary structure of the arm. Although variation occurs primarily in the DHU and TΨC arms, most changes are observed in the loops, while the stems are more conserved, as has been observed in other ant subfamilies as well as in other insect groups [17,64]. The stem regions form double-stranded structures that are critical for the stability of the tRNA’s secondary structure, which is why they tend to be more conserved. In fact, the observed changes in these regions do not affect the stem formation. When substitutions do occur, they lead to A-U to G-U pairings or vice versa, as seen in the TΨC arms of tRNA-Met and tRNA-Gly (Figure 5). Small insertions or deletions have also been observed in the stems of these two arms, yet the secondary structure of the tRNAs does not appear to be affected (Supplementary Figure S1).

The mitogenomes of *Tapinoma* show the placement of the large ribosomal RNA subunit gene (*lrRNA*) between the *tRNA-Leu* and *tRNA-Val* genes. It is assumed that the bases between these two genes comprise the *lrRNA* gene. According to this, the 3′ end of the small ribosomal RNA subunit gene (*srRNA*) would be delimited by the presence of the *tRNA-Val* gene. However, no tRNA gene flanks the 5′ end of the *srRNA* gene. To determine the location of the *srRNA* gene, we used the annotation provided by the MITOS2 software, which considers the secondary structure for the annotation [26], along with comparisons to previously described *Tapinoma* mitogenomes and those of other Dolichoderinae species. In agreement with the obtained results, the lengths of the *lrRNA* and *srRNA* genes in the TNC mitogenomes described in this study are very similar to those in the *T. ibericum* mitogenome (1345 and 791 bp, respectively) (Supplementary Table S1). The *lrRNA* lengths range from 1344 bp in *T. darioi* to 1347 bp in *T. hispanicum*, and the length of the *srRNA* gene ranges from 791 bp in *T. darioi* to 798 bp in *T. nigerrimum*. Furthermore, the mitogenomes of species outside this complex are also comparable, with *T. simrothi* showing lengths of 1343 and 795 bp, respectively, and *T. madeirense* displaying slightly higher values of 1356 and 799 bp, respectively. Regarding the total A + T content in these genes, all mitogenomes exhibit a similar average, 88%, for the *lrRNA* gene and 89% for the *srRNA* gene, consistent with the genome of *T. ibericum* [16].

Among the non-coding fragments in the mitogenome, the CR is crucial since it is responsible for the initiation of mtDNA transcription and replication. In analyzed insect mitogenomes, certain patterns related to the CR have been observed: it is the largest non-coding sequence, highly variable, with the possible presence of tandem internal repeats and extraordinarily high A + T content [65]. Similarly, a wide diversity in the CR has been described in different ant species, suggesting that it may be associated with adaptive evolution to the heterogeneous habitats of this group of insects [17,66]. The CR is one of the most difficult regions to identify using both traditional methods and NGS, primarily due to the high sequence variability and the presence of internal repeats [57,67]. The CRs of the *Tapinoma* mitogenomes described in this study exhibit an A + T richness of approximately 99% in all cases and lack internal repeats. Despite the heterogeneity in the size and organization of the CRs, the existence of conserved sequences that could form a stem–loop configuration necessary for the initiation of the mitogenome replication has been suggested [68]. The analysis of the potential secondary structure of the CRs in the analyzed *Tapinoma* mitogenomes shows the presence of inverted repeats in all of them (Supplementary Figure S2), which could lead to the formation of these stem–loop secondary structures, some of which could act as possible replication origins.

The results of this study, based on the analysis of the mitogenome of *Tapinoma* species, confirm the previously established relationships based on morphological character and microsatellite data, highlighting the utility of mitochondrial DNA sequences in such research. Its maternal inheritance, high mutation rate, and the presence of conserved regions allow for the precise resolution of evolutionary relationships at both intraspecific and interspecific levels. Additionally, its non-recombinant nature provides a complementary and reliable tool to validate and strengthen evolutionary hypotheses, demonstrating its value in comparative phylogenetic studies, especially when used in conjunction with other molecular markers such as those derived from nuclear sequences.

## Figures and Tables

**Table 1:** Samples of the *Tapinoma* species whose mitogenomes were sequenced and annotated in this study.

Species Name	Coordinates	Locality Nest	Collection Date
*T. darioi*	40.65401° N, 0.411551° E	Benicarló (Spain)	20 September 2019
*T. magnum*	46.20167° N, 4.828° E	Lyon (France)	2011
*T. hispanicum*	37.7718° N, 3.4972° W	Torres (Spain)	31 October 2014
*T. nigerrimum*	43.684254° N, 3.876259° E	Prades-le-Lez (France)	30 April 2012
*T. simrothi*	37.714281° N, 3.904023° W	Jamilena (Spain)	23 April 2014
*T. madeirense*	37.259167° N, 3.487777° W	Sierra de Huetor (Spain)	19 April 2004

**Table 2:** Available mitochondrial genome sequences so far for Dolichoderinae.

Species	Mitogenome Size (bp)	Country of Origin	Accession Number	Reference
Tribe Dolichoderini				
*Dolichoderus lamellosus* (Mayr, 1870)	16,234	Costa Rica	BK012125	[30]
*Dolichoderus pustulatus* (Mayr, 1886)	16,224	Canada	BK012668	[30]
*Dolichoderus quadripunctatus* (Linnaeus, 1771)	16,017	Poland	MT178447	[31]
*Dolichoderus sibiricus* (Emery, 1889)	16,086	South Korea	MH719017	[32]
	16,044	South Korea	MK801110	[31]
	16,067	Russia	MT919976	unpublished
	16,110	Taiwan	MW160468	unpublished
Tribe Leptomyrmecini				
*Dorymyrmex brunneus* (Forel, 1908)	15,848	-	MG253267	unpublished
*Leptomyrmex erythrocephalus* (Fabricius, 1775)	15,546	Australia	BK012481	[30]
*Leptomyrmex pallens* (Emery, 1883)	15,591	New Calcedonia	KC160533	[33]
*Linepithema humile* (Mayr, 1868)	16,098	USA	KT428891	[34]
	15,929	-	KX146468	[35]
	15,934	South Korea	MT890564	[36]
*Ochetellus glaber* (Mayr, 1862)	16,259	South Korea	MN044390	[37]
Tribe Tapinomini				
*Tapinoma darioi* (Seifert et al., 2017)	15,680	Spain	PQ459328	This study
*Tapinoma hispanicum* (Seifert et al., 2024)	15,665	Spain	PQ459329	This study
*Tapinoma ibericum* (Santschi, 1925)	15,715	Spain	NC_065783	[16]
*Tapinoma madeirense* (Forel, 1895)	15,507	Spain	PQ459330	This study
*Tapinoma magnum* (Mayr, 1861)	15,694	France	PQ459331	This study
*Tapinoma melanocephalum* (Fabricius, 1793)	15,499	China	MN397938	[38]
*Tapinoma nigerrimum* (Nylander, 1856)	15,817	France	PQ459332	This study
*Tapinoma sessile* (Say, 1836)	15,287	USA	BK012786	[30]
*Tapinoma simrothi* (Krausse, 1911)	15,487	Spain	PQ459333	This study

**Table 3:** Start and stop codons in each PCG in the mitogenomes of *Tapinoma* species. Same start codons share the blue (ATA), green (ATG), brown (ATT), and yellow (ATC) background. Incomplete stop codons share the green (TA-), and blue (T--) background.

	***Start codons***												
	*nad2*	*cox1*	*cox2*	*atp8*	*atp6*	*cox3*	*nad3*	*nad5*	*nad4*	*nad4l*	*nad6*	*cob*	*nad1*
*T. nigerrimum*	ATA	ATG	ATT	ATC	ATG	ATG	ATA	ATA	ATG	ATT	ATG	ATG	ATT
*T. darioi*	ATA	ATG	ATT	ATC	ATG	ATG	ATA	ATA	ATG	ATT	ATG	ATG	ATT
*T. hispanicum*	ATA	ATG	ATT	ATC	ATG	ATG	ATA	ATA	ATG	ATT	ATG	ATG	ATT
*T. ibericum*	ATA	ATG	ATT	ATC	ATG	ATG	ATA	ATA	ATG	ATT	ATG	ATG	ATT
*T. magnum*	ATA	ATG	ATT	ATT	ATG	ATG	ATA	ATA	ATG	ATT	ATG	ATG	ATT
*T. simrothi*	ATA	ATG	ATT	ATA	ATG	ATG	ATA	ATA	ATG	ATT	ATG	ATG	ATT
*T. madeirense*	ATA	ATG	ATT	ATA	ATG	ATG	ATT	ATT	ATG	ATT	ATG	ATG	ATA
*T. sessile*	ATA	ATG	ATT	ATA	ATG	ATG	ATT	ATT	ATG	ATT	ATG	ATG	ATT
*T. melanocephalum*	ATA	ATG	ATT	ATT	ATG	ATG	ATA	ATA	ATG	ATT	ATT	ATG	ATT
	***Stop codons***												
	*nad2*	*cox1*	*cox2*	*atp8*	*atp6*	*cox3*	*nad3*	*nad5*	*nad4*	*nad4l*	*nad6*	*cob*	*nad1*
*T. nigerrimum*	TAA	TAA	TAA	TAA	TAA	TAA	TAA	TAA	TAA	TAA	TAA	TAA	TAA
*T. darioi*	TAA	TAA	TAA	TAA	TAA	TAA	TAA	TAA	TAA	TAA	TAA	TAA	TAA
*T. hispanicum*	TAA	TAA	TAA	TAA	TAA	TAA	TAA	TAA	TAA	TAA	TAA	TAA	TAA
*T. ibericum*	TAA	TAA	TAA	TAA	TAA	TAA	TAA	TAA	TAA	TAA	TAA	TAA	TAA
*T. magnum*	TAA	TAA	TAA	TAA	TAA	TAA	TAA	TAA	TAA	TAA	TAA	TAA	TAA
*T. simrothi*	TA-	TA-	TAA	TAA	TAA	TAA	TAA	TA-	TAA	TAA	TAA	TAA	TAA
*T. madeirense*	TAA	TA-	TAA	TAA	TAA	TAA	TAA	TAA	TAA	TAA	TAA	TAA	TAA
*T. sessile*	TAA	TA-	TAA	TAA	TAA	TAA	TAA	TA-	TAA	TAA	TAA	TAA	TAA
*T. melanocephalum*	T--	TA-	TAA	TAA	TAA	TAA	TAA	T-	TAA	TAA	TAA	TAA	TAA

## Data Availability

The *Tapinoma* mitogenome sequences have been submitted to the NCBI database (acc. Numbers PQ459328- PQ459333).

## Supplementary Materials

The following supporting information can be downloaded at https://www.mdpi.com/article/10.3390/insects15120957/s1: Figure S1: Alignment of tRNA sequences from the mitogenomes of *Tapinoma* species and secondary structure. Figure S2: Alignment of the control regions in *Tapinoma* species and potential secondary structure. Table S1: Mitogenome annotations in *Tapinoma* species. Table S2: Codon usage in *Tapinoma* species.
