# Supplementary material for: A Comparative Analysis of Mitogenomes in Species of the Tapinoma nigerrimum Complex and Other Species of the Genus Tapinoma (Formicidae, Dolichoderinae)
Source: Insects. 2024 Dec 2;15(12):957. doi: 10.3390/insects15120957 (PMC11677639; doi:10.3390/insects15120957)

Figure S1. Alignment of tRNA sequences from the mitogenomes of *Tapinoma* species within the *Tapinoma nigerrimum* complex, *T. simrothi* and *T. madeirense*, and secondary structures.

tRNA-Met

```
                              10        20        30        40        50        60        70
                      ....|....|....|....|....|....|....|....|....|....|....|....|....|....|
Tapinoma ibericum     AAATAAGTAAGCTAA-AAT-AAGCTTTTAGGTTCATACCCTAAATATAGGTTTTAAA----AACCTCTTATTTA 68
Tapinoma hispanicum   ...............-...T.....................................-----..T......... 69
Tapinoma nigerrimum   ...............T...T.....................................ATTT-..T......... 74
Tapinoma darioi       ...............T.-.T.....................................ATTT-..T......... 73
Tapinoma magnum       ...............T..--.....................................-TTT-............ 71
Tapinoma simrothi     ...............T--.T.............................A..A....ATTTT..T......... 73
Tapinoma madeirense   .TT............-.T.T.............................A.......AT------......... 68
```

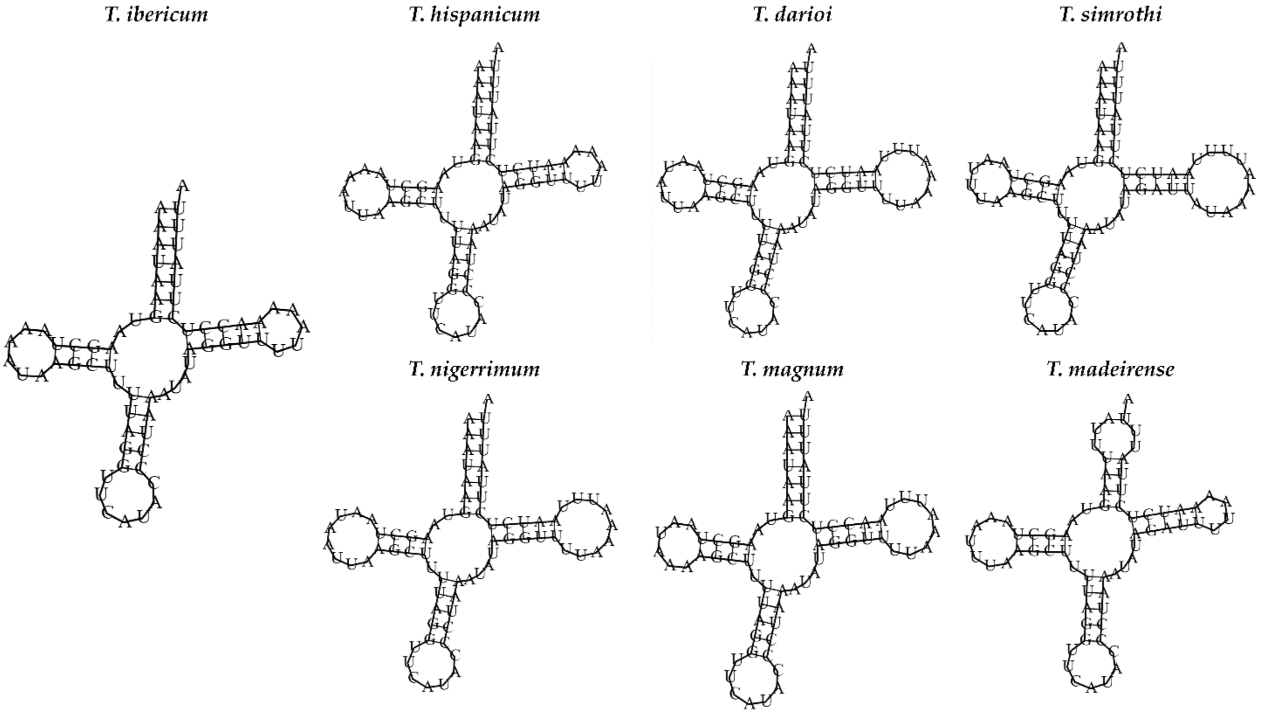

tRNA-Ile

```
                                10        20        30        40        50        60        70
                       ....|....|....|....|....|....|....|....|....|....|....|....|....|....|...
Tapinoma iberium       AATGAAATGCCTGATAAAAGGATTATTTTGATAGAATAAATTATACAT--ATTTTTAT-----GTTTTCATTA 66
Tapinoma hispanicum    ................................................--........-----.......... 66
Tapinoma nigerrimum    ................................................--........-----.......... 66
Tapinoma darioi        ................................................--........-----.......... 66
Tapinoma magnum        ................................................--........TTTAT.......... 71
Tapinoma simrothi      ...............T................................-A........-----.......... 67
Tapinoma madeirense    ................................................AA........-----.......... 68
```

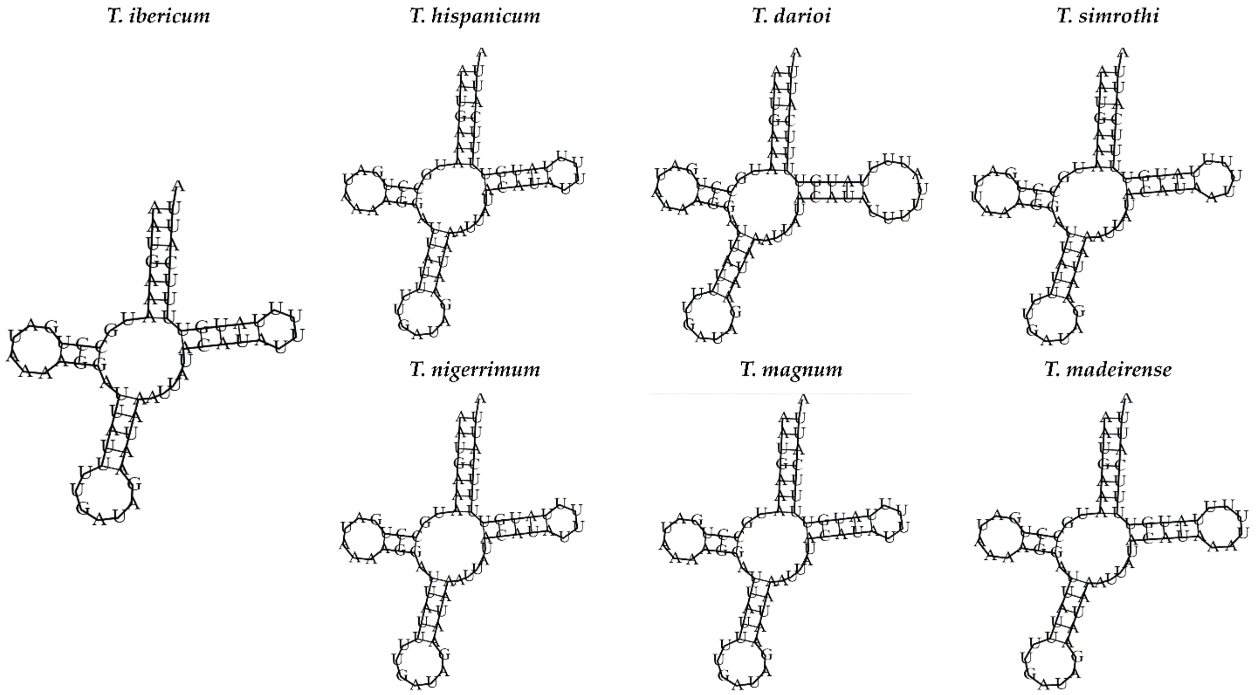

tRNA-Gln

```
                           10        20        30        40        50        60        70
                   ....|....|....|....|....|....|....|....|....|....|....|....|....|....|
Tapinoma ibericum      TATATTTTAATGTAT-GAAGCATAATAATATTTGAAATTATAAGAAAAAGTTTAATTCTTTTAAATATAA 69
Tapinoma hispanicum    ...............-...................................................... 69
Tapinoma nigerrimum    ...............-...................................................... 69
Tapinoma darioi        ...............-...................................................... 69
Tapinoma magnum        ...............-...................................................... 69
Tapinoma simrothi      ...............-...................................................... 69
Tapinoma madeirense    ..............AA.......G.............................G................ 70
```

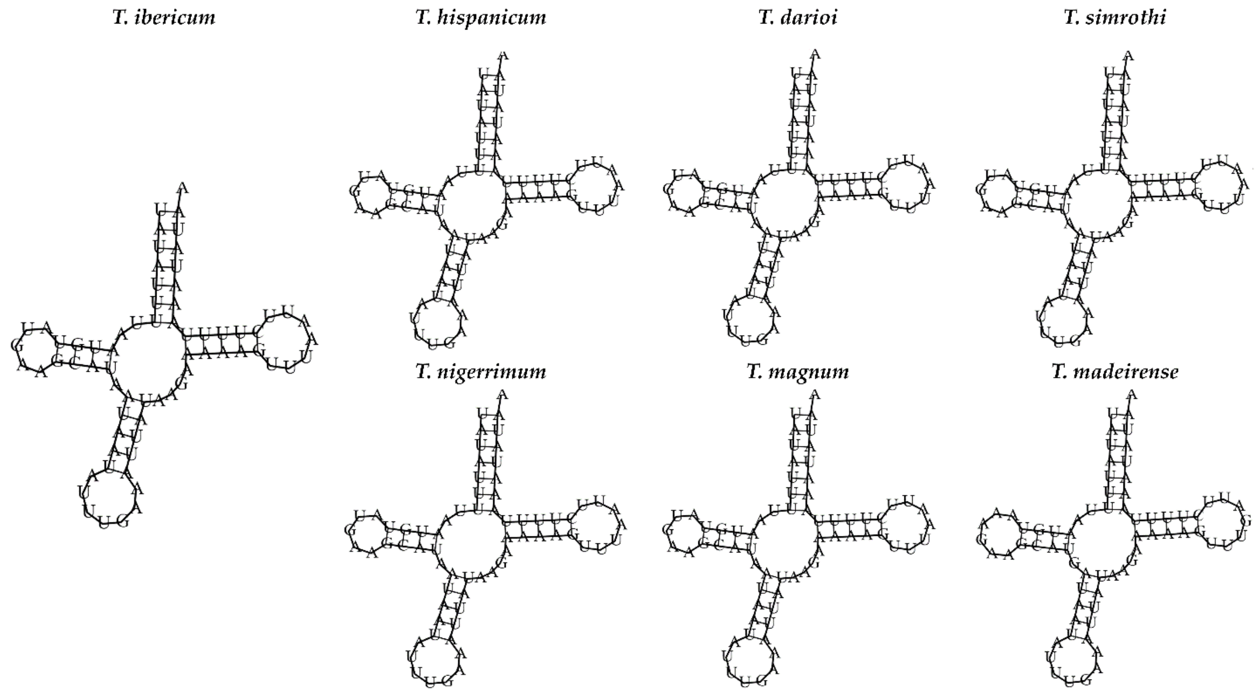

tRNA-Trp

```
                                  10        20        30        40        50        60        70
                          ....|....|....|....|....|....|....|....|....|....|....|....|....|....|....|
Tapinoma ibericum         AAGATTTTAAGTTAT-TTTAAACTTTAAACCTTCAAAGTTTAATAAATATATTAAATAAATTTATATAAATCTTA 74
Tapinoma hispanicum       ...............-........................................................... 74
Tapinoma nigerrimum       ...............-........................................................... 74
Tapinoma darioi           ...............-........................................................... 74
Tapinoma magnum           ...............-........................................................... 74
Tapinoma simrothi         ...............T.............................--.....A.T.................... 73
Tapinoma madeirense       ...............-.............................--.....A..--..T.C............. 70
```

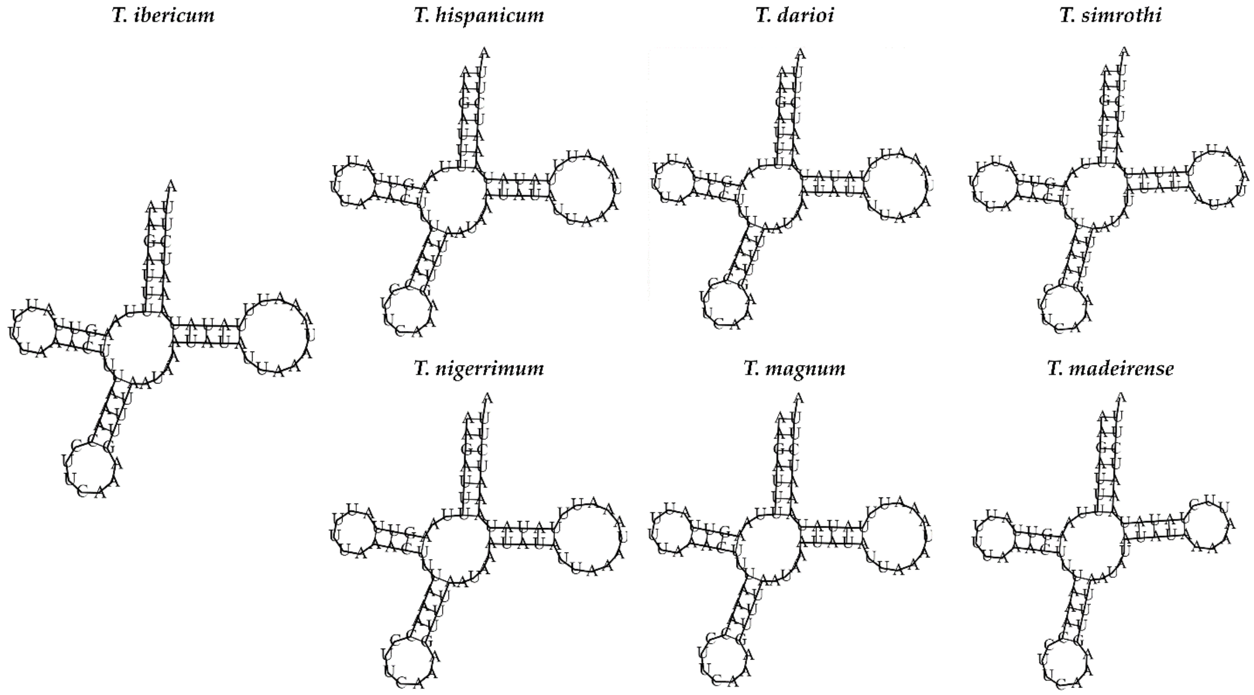

tRNA-Tyr

```
                                 10        20        30        40        50        60
                        ....|....|....|....|....|....|....|....|....|....|....|....|....|....
Tapinoma ibericum       AGTATTATGTCTGATTTAAAAGTAATAAATTGTAAATTTATTTATGAAAATTTTATTTTTCTAATACTA 69
Tapinoma hispanicum     ..................................................................... 69
Tapinoma nigerrimum     ..................................................................... 69
Tapinoma darioi         ..................................................................... 69
Tapinoma magnum         ..................................................................... 69
Tapinoma simrothi       ...................................................-................. 68
Tapinoma madeirense     ...................................................-...A............. 68
```

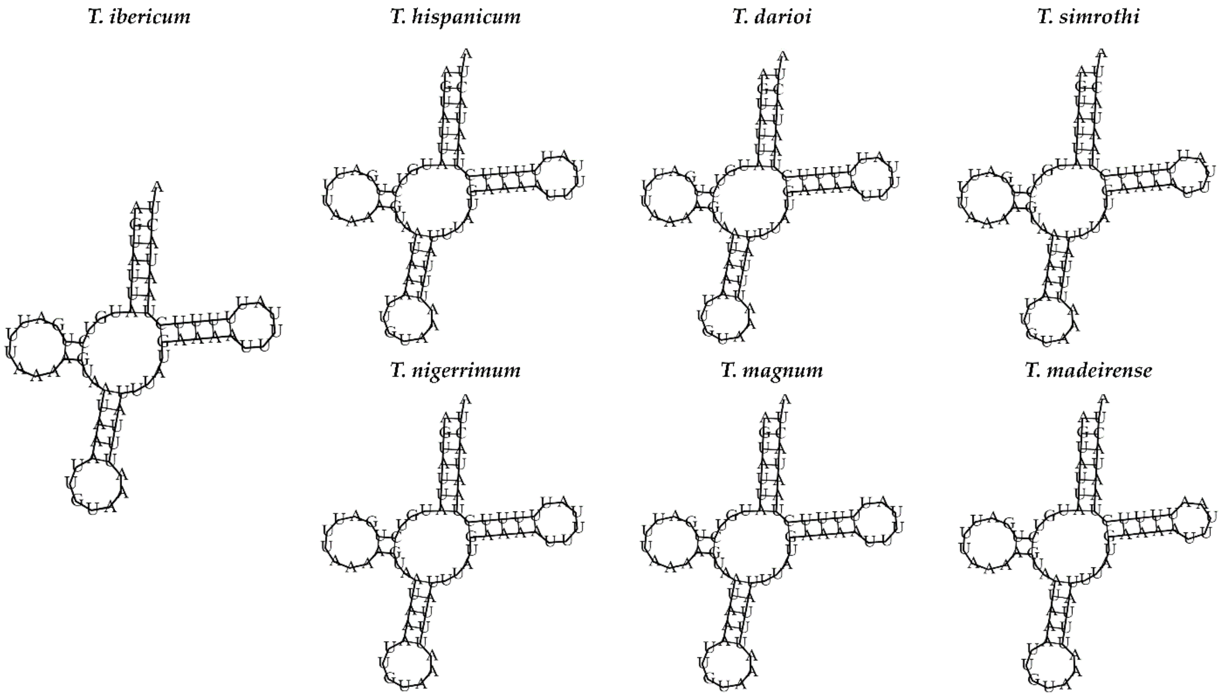

tRNA-Cys

```
                              10        20        30        40        50        60
                      ....|....|....|....|....|....|....|....|....|....|....|....|....|....
Tapinoma iberticum    TATTTTATAGTTTATT-ATAAAACATTAAATTGCAAATTTAAAGATATTAAT-AAAATAATTAAAATAT 67
Tapinoma hispanicum   ................-...........G.......................-................ 67
Tapinoma nigerrimum   ................-...................................-................ 67
Tapinoma darioi       ................-...................................-................ 67
Tapinoma magnum       ................T.................................................... 69
Tapinoma simrothi     ................-.-....T.....................A....T.T..T............. 67
Tapinoma madeirense   ...............--.A.................G.....TA.A....T.T................ 67
```

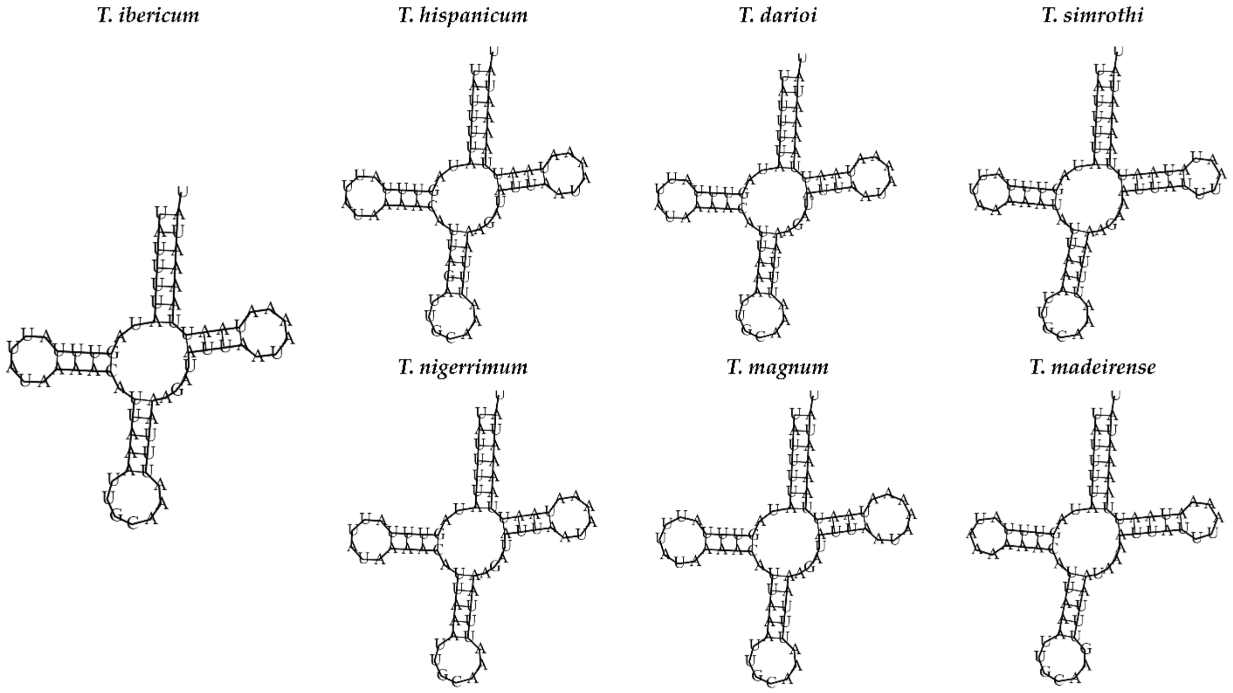

tRNA-Leu (1, UUR)

```
                               10        20        30        40        50        60
                          ....|....|....|....|....|....|....|....|....|....|....|....|....|...
Tapinoma ibericum         TTTAATATGGCAGATTAGTGCAATGAATTTAAACTTCATTTATAAAGA--TTTTTTCTTTTATTAAAA 66
Tapinoma hispanicum       ................................................TT.................. 68
Tapinoma nigerrimum       ................................................TT.................. 68
Tapinoma darioi           ................................................--.................. 66
Tapinoma magnum           ................................................--.................. 66
Tapinoma simrothi         ................................................---................. 65
Tapinoma madeirense       .................................T........A.....-T..C............... 67
```

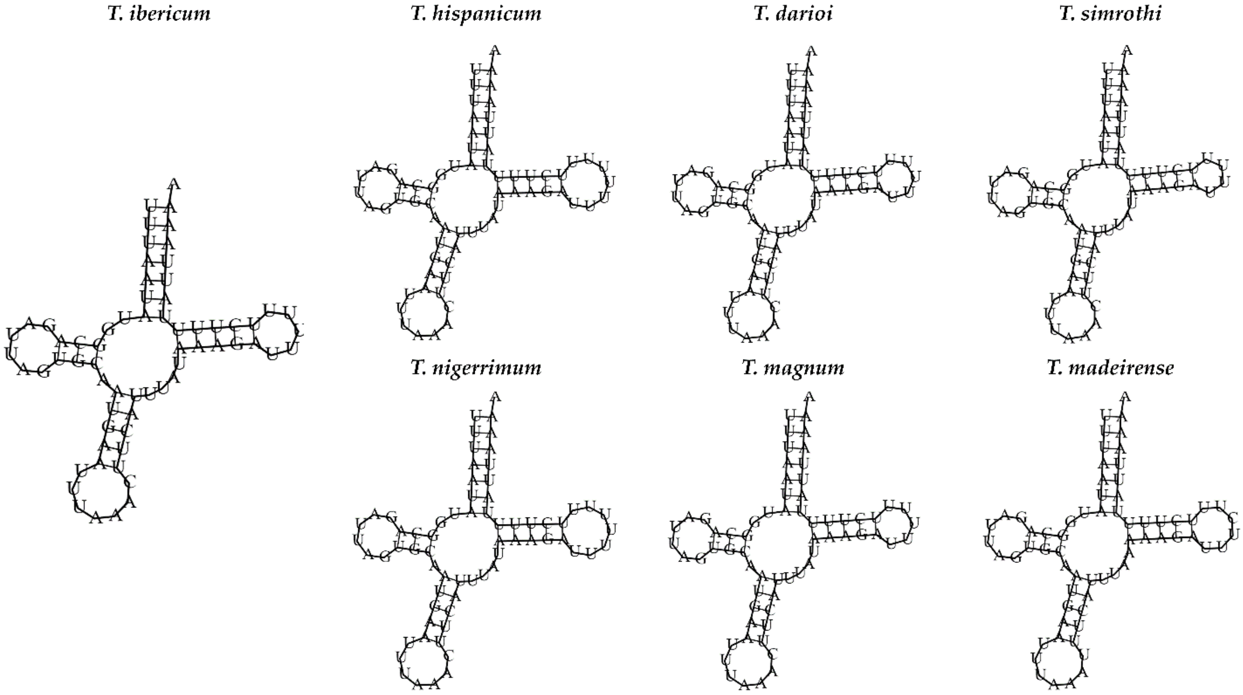

tRNA-Lys

```
                                  10        20        30        40        50        60        70
                         ....|....|....|....|....|....|....|....|....|....|....|....|....|....|...
Tapinoma ibericum        CATTAAATGACCGAAATGTAAGTATTGATCTTTTAAATCAATTATAATAGAGATTAACTTCTATTTTTAATGA 73
Tapinoma hispanicum      ......................................................................... 73
Tapinoma nigerrimum      ......................................................................... 73
Tapinoma darioi          ......................................................................... 73
Tapinoma magnum          ......................................................................... 73
Tapinoma simrothi        .................................................--.......C.............. 71
Tapinoma madeirense      ......................................................................... 73
```

T. ibericum

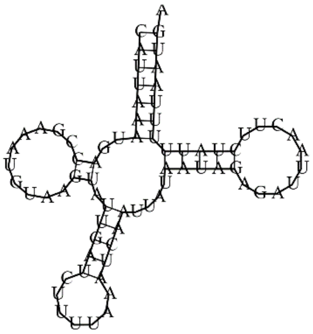

T. hispanicum

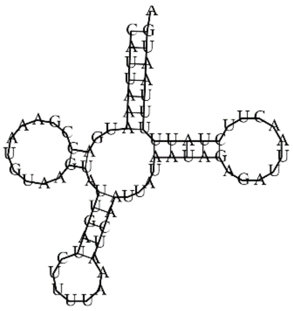

T. darioi

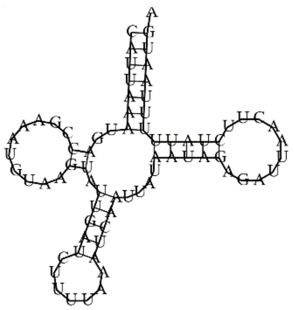

T. simrothi

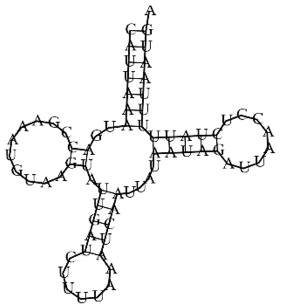

T. nigerrimum

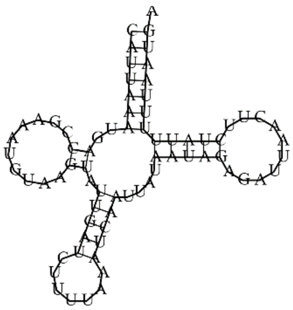

T. magnum

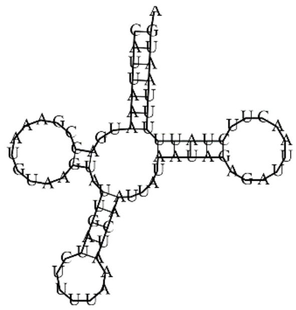

T. madeirense

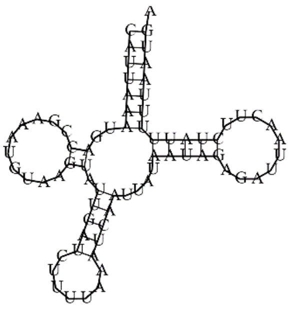

tRNA-Asp

```
                                 10        20        30        40        50        60
                        ....|....|....|....|....|....|....|....|....|....|....|....|....|....
Tapinoma ibericum       AAAAAATTAGTTAAATTATATAATATTAAAATGTCATTTTAAAGTTATTTTATTAAAAAATATTTTTTT 69
Tapinoma hispanicum     -...................................................................- 67
Tapinoma nigerrimum     ..................................................................... 69
Tapinoma darioi         ......................................................-.............. 68
Tapinoma magnum         ......................................................-.............. 68
Tapinoma simrothi       ..................................................-...-.............. 67
Tapinoma madeirense     -..............--......C...................A.....AA...T.TT..........- 65
```

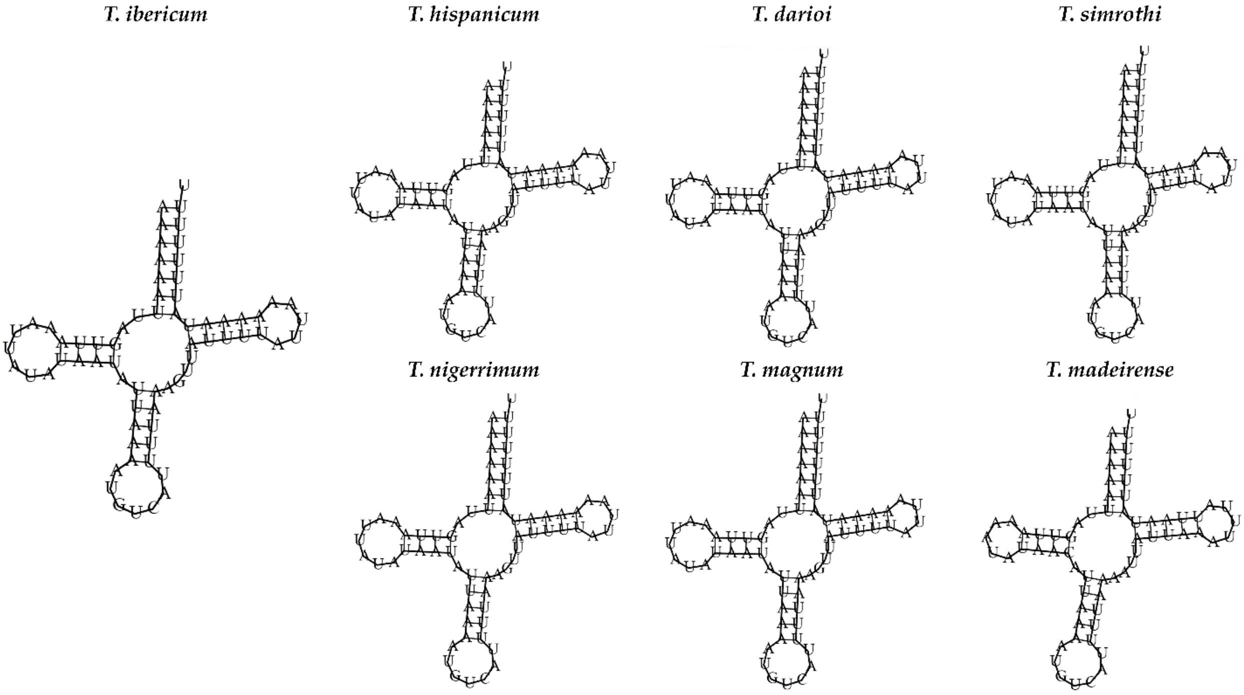

tRNA-Gly

```
                                10        20        30        40        50        60        70
                        ....|....|....|....|....|....|....|....|....|....|....|....|....|....|
Tapinoma ibericum       ATTTATATAGTAT--AAAATATTACAATTAATTTCCAATTAAAAAATTTAAATAACTTTTAATATAAATA 68
Tapinoma hispanicum     .............--..........................................-...G........ 67
Tapinoma nigerrimum     .............A-........................................-.A............ 68
Tapinoma darioi         .............A-........................................-.C............ 68
Tapinoma magnum         .............A-..........................................-............ 68
Tapinoma simrothi       .............--........................................-.............. 67
Tapinoma madeirense     .............AA...........T.......................T....-.A-........... 68
```

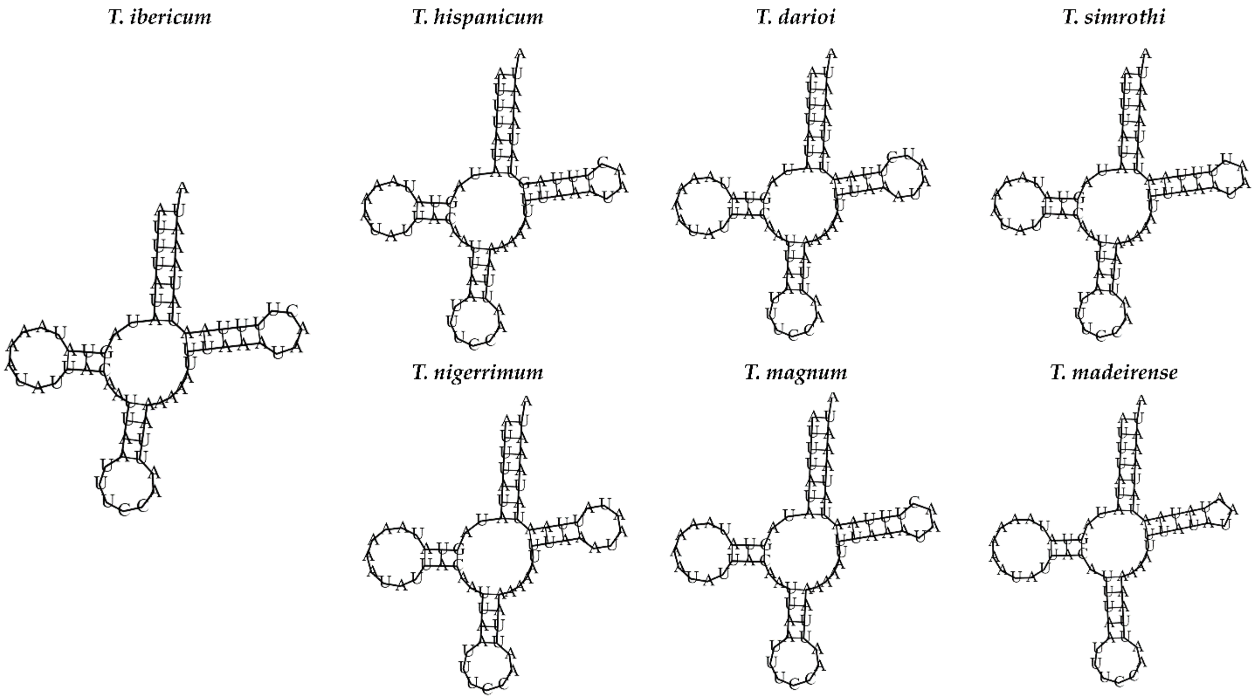

tRNA-Ala

```
                              10        20        30        40        50        60        70
                     ....|....|....|....|....|....|....|....|....|....|....|....|....|....|..
Tapinoma ibericum    TGGATATTAGTT-AAATT-TTTATAACATTTAAATTGCATTTAAAAATTATATTTTATTTAAAA--TATATGTCTAA 73
Tapinoma hispanicum  ............-.....-.............................................--.....A..... 73
Tapinoma nigerrimum  ............-.....-.............................C...............A-.....A..... 74
Tapinoma darioi      ............-.....-.............................................A-.....A..... 74
Tapinoma magnum      ............-.....-.............................C...............AG.....A..... 75
Tapinoma simrothi    ............A.....A..................................----.A....CAT.....A..... 73
Tapinoma madeirense  -...........-.....A........................................A--C.CA-.....A...T- 71
```

T. ibericum

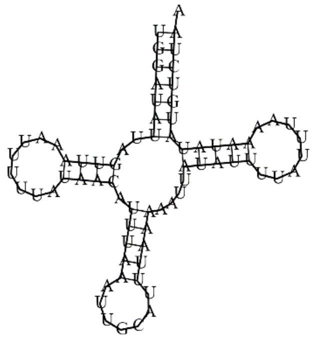

T. hispanicum

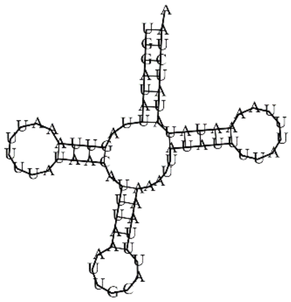

T. darioi

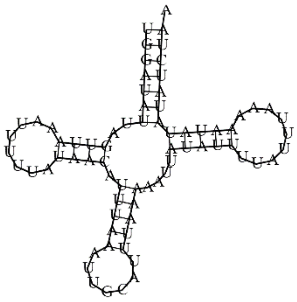

T. simrothi

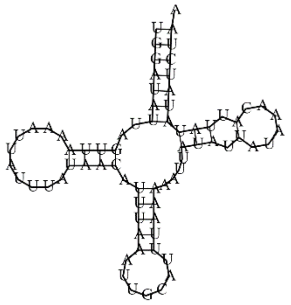

T. nigerrimum

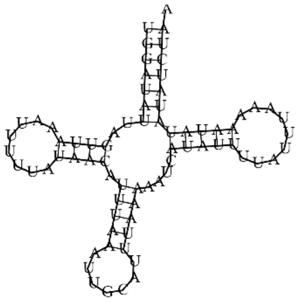

T. magnum

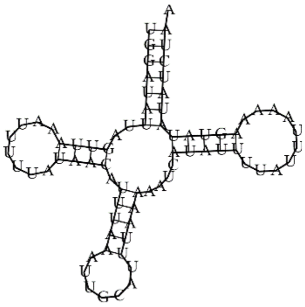

T. madeirense

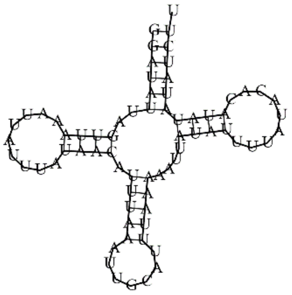

tRNA-Arg

```
                                 10        20        30        40        50        60        70
                     ....|....|....|....|....|....|....|....|....|....|....|....|....|....|....|..
Tapinoma ibericum    ATTCTTAAAGTAATTTATTACATTTAATTTCGACTTAAAAATAGATTATAATATAAAAAAAAATTAATCTAAGAATT 76
Tapinoma hispanicum  ......................................................-..................... 75
Tapinoma nigerrimum  ......................................................-.T................... 75
Tapinoma darioi      ......................................................-.T................... 75
Tapinoma magnum      ......................................................--.................... 74
Tapinoma simrothi    ...........T.....A.......................A..........---------A.............. 67
Tapinoma madeirense  .............AA.T.........................T....-....T----..T................ 71
```

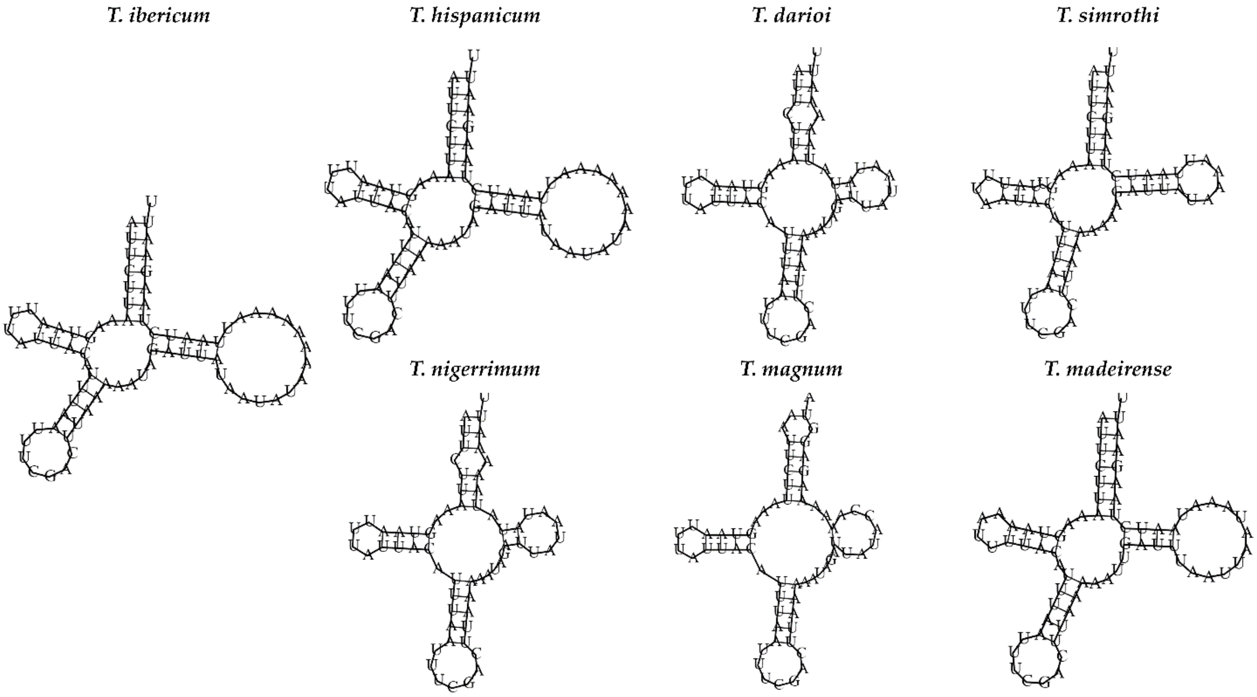

tRNA-Asn

```
                                 10        20        30        40        50        60        70
                        ....|....|....|....|....|....|....|....|....|....|....|....|....|....|....|
Tapinoma ibericum       TTAACTAAAACCAAAAAAGAGGTAAATTATTGTTAATAATTTTATTGAATTA--TCAAAA----TTCTAGTTAAT 69
Tapinoma hispanicum     ....................................................--......----........... 69
Tapinoma nigerrimum     ....................................................--......A---........... 70
Tapinoma darioi         ....................................................--......A---........... 70
Tapinoma magnum         ....................................................AT.AT...AAAA........... 75
Tapinoma simrothi       ....................................................TT.A....A---........... 72
Tapinoma madeirense     ..............-..................................A..--.A..T.AATT........... 72
```

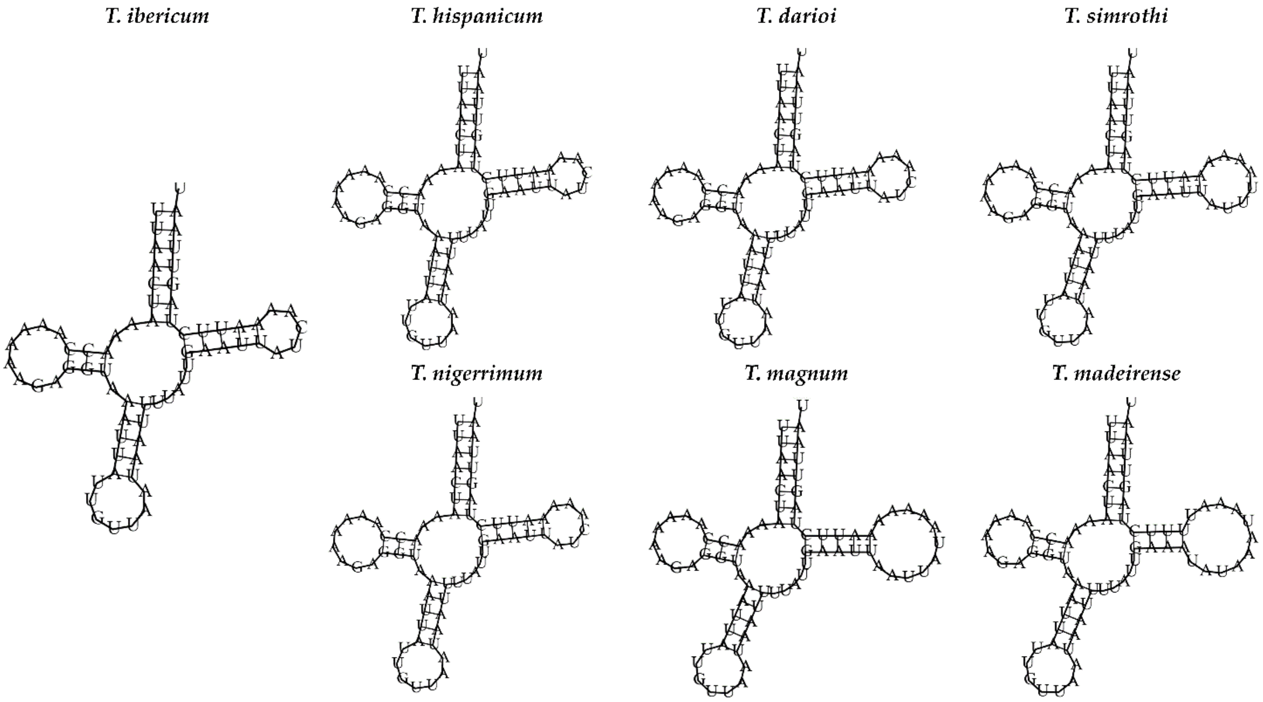

tRNA-Ser (1, AGN)

```
                             10        20        30        40        50        60
                    ....|....|....|....|....|....|....|....|....|....|....|....|....|
Tapinoma ibericum   GATATATTTTAAATTGAACTTCTAATTCAATATTAGTGATTTTAA--TTTTAATCACATATATTT 63
Tapinoma hispanicum .............................................--.................. 63
Tapinoma nigerrimum .............................................AA.................. 65
Tapinoma darioi     .............................................AA.................. 65
Tapinoma magnum     .............................................--.................. 63
Tapinoma simrothi   .........A...............................A...----..T............. 61
Tapinoma madeirense ................................C......-.A..------.T............. 58
```

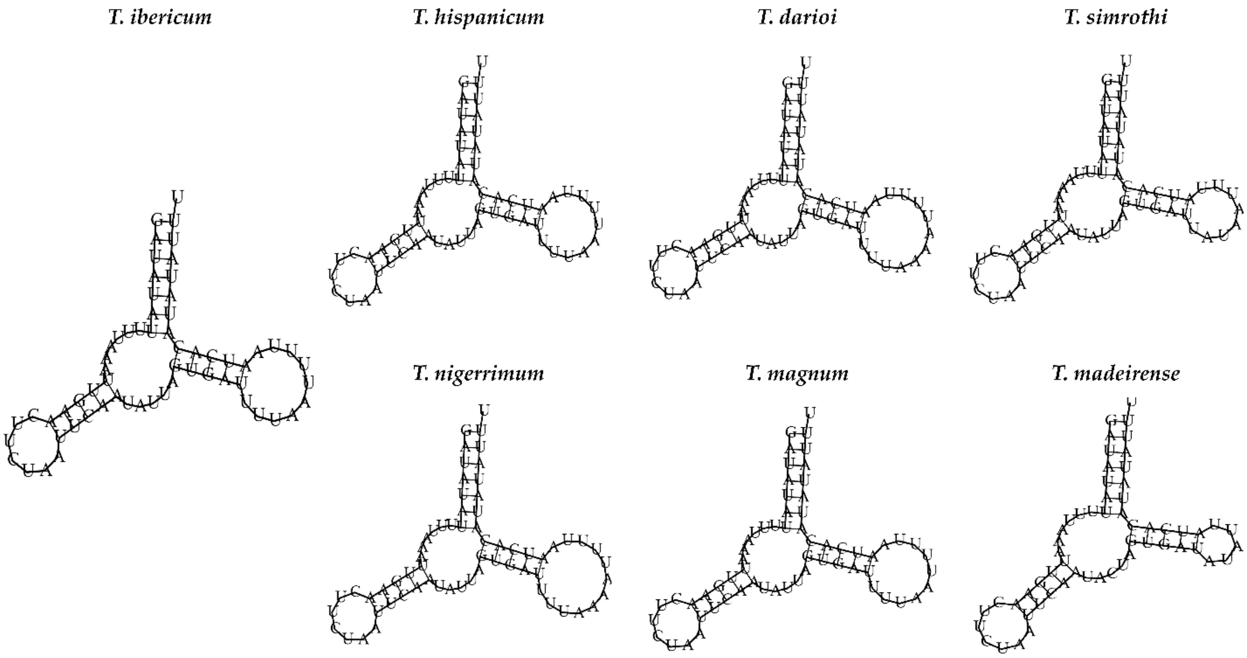

tRNA-Glu

```
                                10        20        30        40        50        60        70
                            ....|....|....|....|....|....|....|....|....|....|....|....|....|....|..
Tapinoma iberricum          TTTATAATAGTTTAAT-ATTTAAAACATTATATTTTCATTATAAAAATATTTCTTTAAAAAAATTTATAAAT 71
Tapinoma hispanicum         ................-....................................................... 71
Tapinoma nigerrimum         ................-....................................................... 71
Tapinoma darioi             ................-....................................................... 71
Tapinoma magnum             ................-....................................................... 71
Tapinoma simrothi           ......-......----...C...............................T..--............... 65
Tapinoma madeirense         ......-........AA...A...............................TA.C.TT............. 71
```

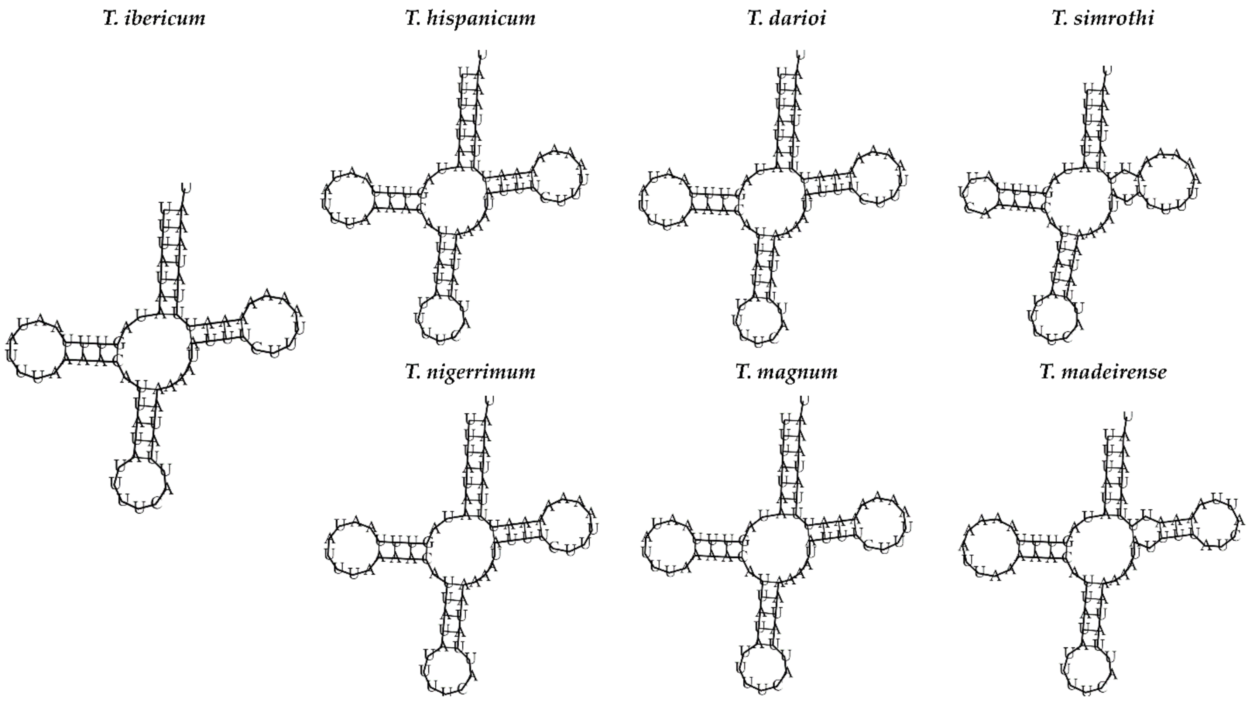

tRNA-Phe

```
                                 10        20        30        40        50        60        70
                             ....|....|....|....|....|....|....|....|....|....|....|....|....|...
Tapinoma ibericum            ATTTAAATAGCTTAAATAAATAAAGTTTAATATTGAAGATATTAAGATAATAT-AATAAAATATTTTTAAATA 72
Tapinoma hispanicum          .....................................................-................... 72
Tapinoma nigerrimum          ...................G.................................--.................. 71
Tapinoma darioi              .....................................................--.A................ 71
Tapinoma magnum              ................-..TA....C...........................GT.................. 72
Tapinoma simrothi            .................-.G......A.................T.......---.................. 69
Tapinoma madeirense          -..............-.G.......CG..................A..G...----A...T...C.......- 66
```

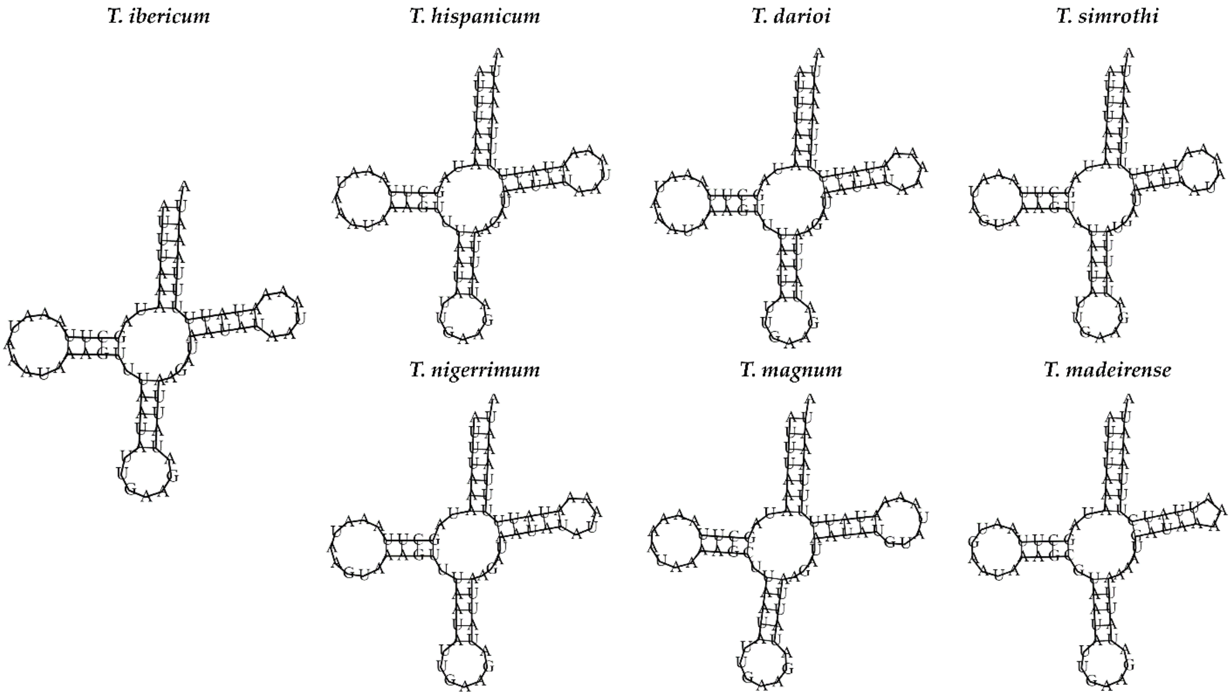

tRNA-His

```
                                      10        20        30        40        50        60        70
                             ....|....|....|....|....|....|....|....|....|....|....|....|....|....|
Tapinoma ibericum            ATTTAAATAGTTTAAT-TTTAAAATAATAATTTGTGATATTATAGATATATTTTTAAAATATTTTAAATC 69
Tapinoma hispanicum          ................-..................................................... 69
Tapinoma nigerrimum          ................-..................................................... 69
Tapinoma darioi              ................-..................................................... 69
Tapinoma magnum              ................-..................................................... 69
Tapinoma simrothi            ................-...................G..............--................T 67
Tapinoma madeirense          ...............AC..................................-A.A..............T 69
```

T. ibericum

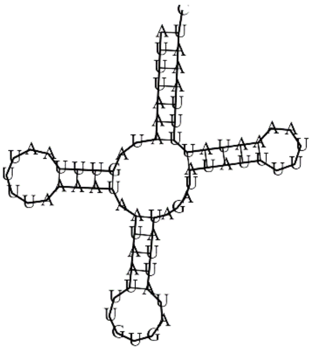

T. hispanicum

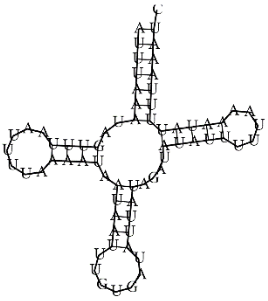

T. darioi

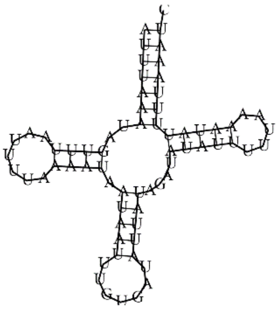

T. simrothi

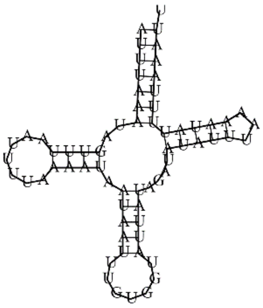

T. nigerrimum

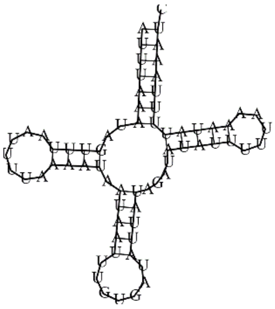

T. magnum

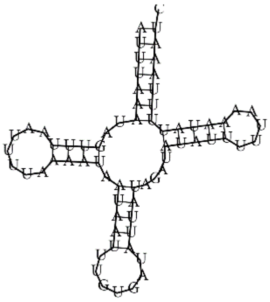

T. madeirense

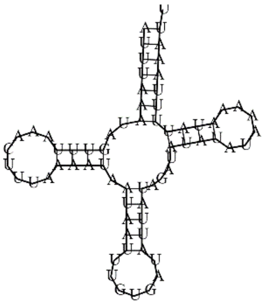

tRNA-Thr

```
                                 10        20        30        40        50        60        70
                         ....|....|....|....|....|....|....|....|....|....|....|....|....|....|....
Tapinoma ibericum        TAGCTTTTAGTTTAATAT---AAAACATAAATTTTGTAAATTTAAAATAACTGAAATTAATCAATTATAGCTAA 71
Tapinoma hispanicum      ...............C..---...............................................G..... 71
Tapinoma nigerrimum      ..................T--..................................................... 72
Tapinoma darioi          ..................T--..................................................... 72
Tapinoma magnum          ..................T-A..................................................... 73
Tapinoma simrothi        ................T.TTA.............................T.A....--..-............ 71
Tapinoma madeirense      ................T----.............................T.----.....-......G....T 65
```

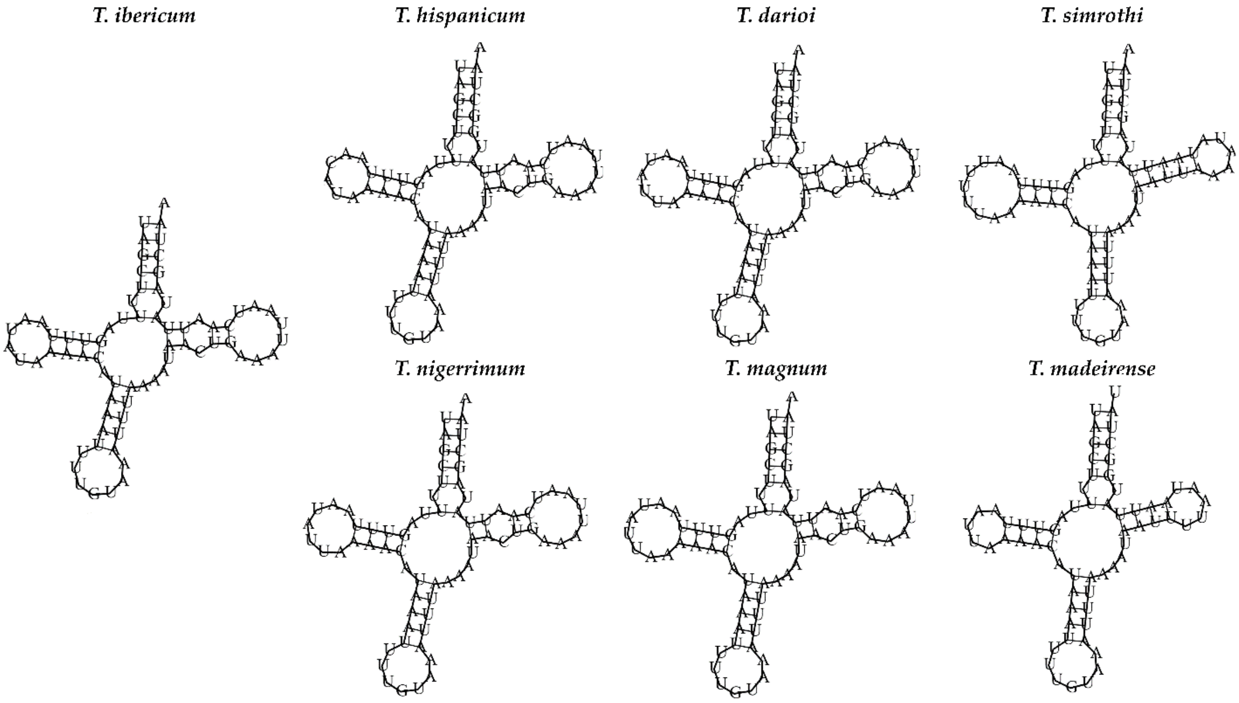

tRNA-Pro

```
                               10        20        30        40        50        60        70
                      ....|....|....|....|....|....|....|....|....|....|....|....|....|....|...
Tapinoma ibericum     CAAAAAATAGTTTA-AAATATATGAATATTAATTTTGGAGATTAAAGGTATAAAATAAATTTTATTTTTTTGA 72
Tapinoma hispanicum   ..............-.......................................................... 72
Tapinoma nigerrimum   ..............-.......................................................... 72
Tapinoma darioi       ..............-.......................................................... 72
Tapinoma magnum       ..............-.......................................................... 72
Tapinoma simrothi     ..............T.......-..............................TT...--............. 70
Tapinoma madeirense   .............T---...A.-..............................TTA....A............ 69
```

T. ibericum

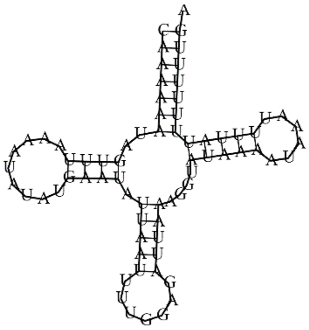

T. hispanicum

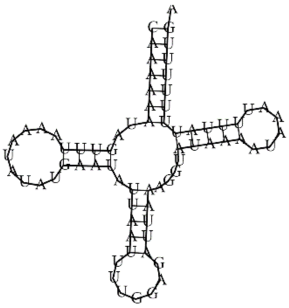

T. darioi

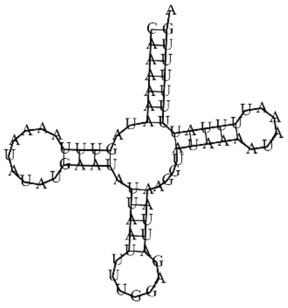

T. simrothi

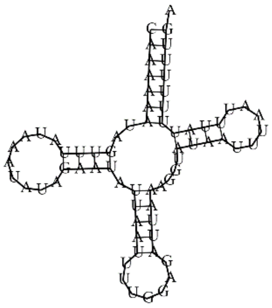

T. nigerrimum

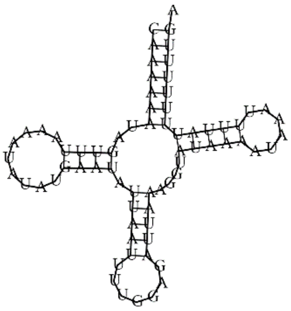

T. magnum

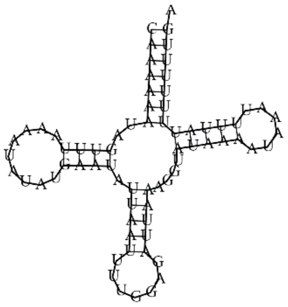

T. madeirense

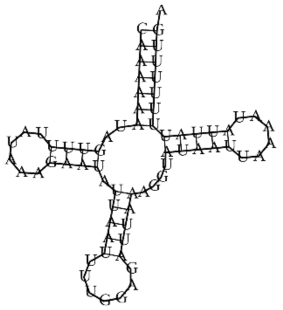

tRNA-Ser (2, UCN)

```
                                10        20        30        40        50        60
                       ....|....|....|....|....|....|....|....|....|....|....|....|....|...
Tapinoma ibericum      AATTAATGATCTTGA-ATAAGAATATGTTTTGAAAACATATTAAAGAAATAAAATTTTCTATTAATTT 67
Tapinoma hispanicum    ...............-.................................................... 67
Tapinoma nigerrimum    ...............-.................................................... 67
Tapinoma darioi        ...............-.................................................... 67
Tapinoma magnum        ...............-.................................................... 67
Tapinoma simrothi      ...............-.................................................... 67
Tapinoma madeirense    ...............T.................................................... 68
```

T. ibericum

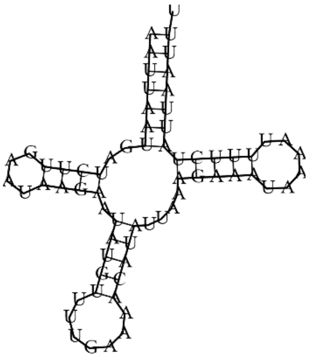

T. hispanicum

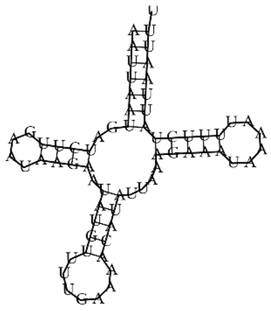

T. darioi

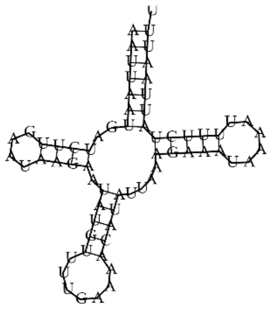

T. simrothi

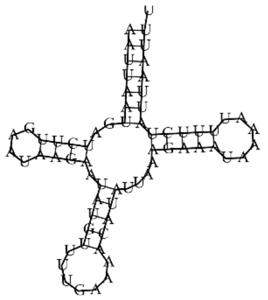

T. nigerrimum

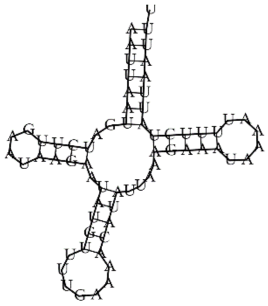

T. magnum

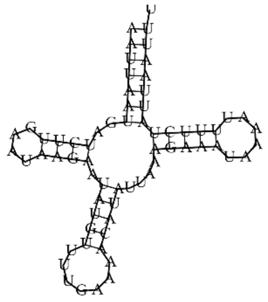

T. madeirense

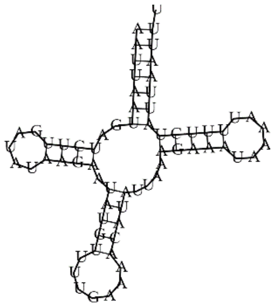

tRNA-Leu (2, CUN)

```
                            10        20        30        40        50        60        70
                   ....|....|....|....|....|....|....|....|....|....|....|....|....|....|...
Tapinoma ibericum   ATTACTTTGGCAGAATAGTGTAATAAATTTAGAATTTATATTAAAATATATAATAAATTATAT-TAAGTAATA 72
Tapinoma hispanicum ........................................................G......-......... 72
Tapinoma nigerrimum ...............................................................-......... 72
Tapinoma darioi     ...............................................................-......... 72
Tapinoma magnum     ...............................................................-......... 72
Tapinoma simrothi   ..............TA...C.T................A............T..TT.A-....G..G...... 72
Tapinoma madeirense ..............T..................G...........-......G.T.---....A......... 69
```

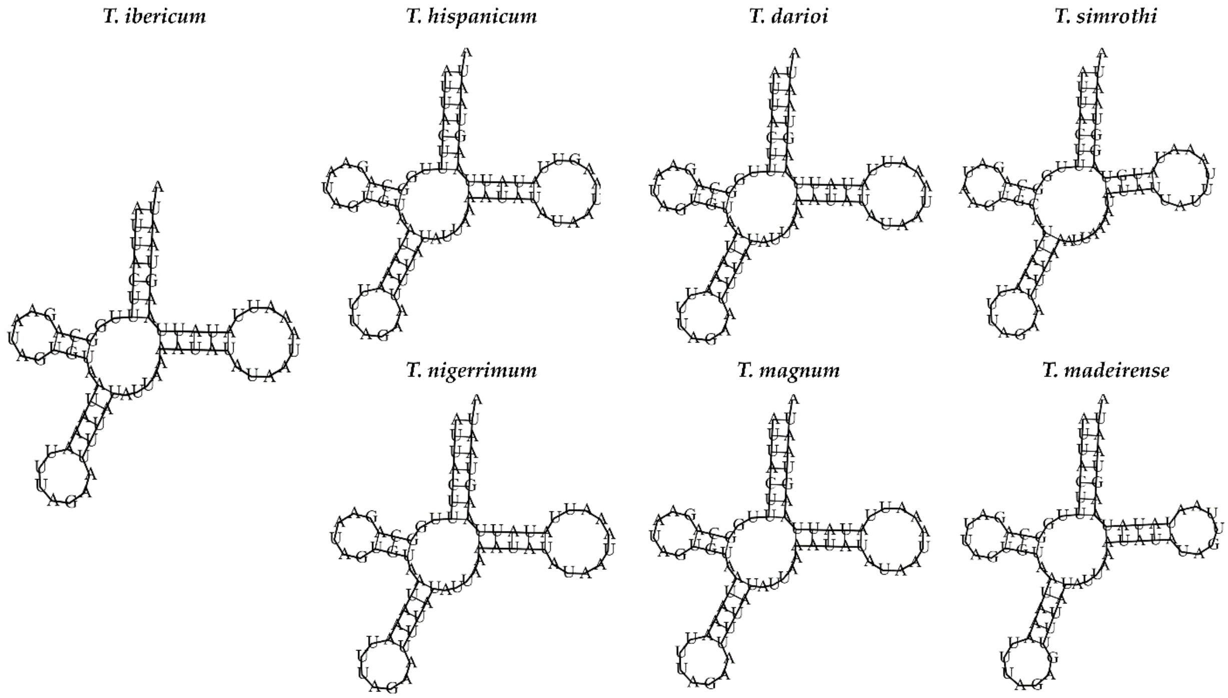

tRNA-Val

```
                                10        20        30        40        50        60        70
                        ....|....|....|....|....|....|....|....|....|....|....|....|....|....|....
Tapinoma ibericum       AAAATTTTAGTTTAATAATTAAAATATTTCATTTACATTGAAAAGTTTATTAATA--TTTATAATAAAATTTTA 72
Tapinoma hispanicum     .......................................................--................. 72
Tapinoma nigerrimum     .......................................................TA................. 74
Tapinoma darioi         .......................................................--................. 72
Tapinoma magnum         .......................................................TT................. 74
Tapinoma simrothi       .......................................................T-................. 73
Tapinoma madeirense     ...................-...............................-G..G-.A............... 71
```

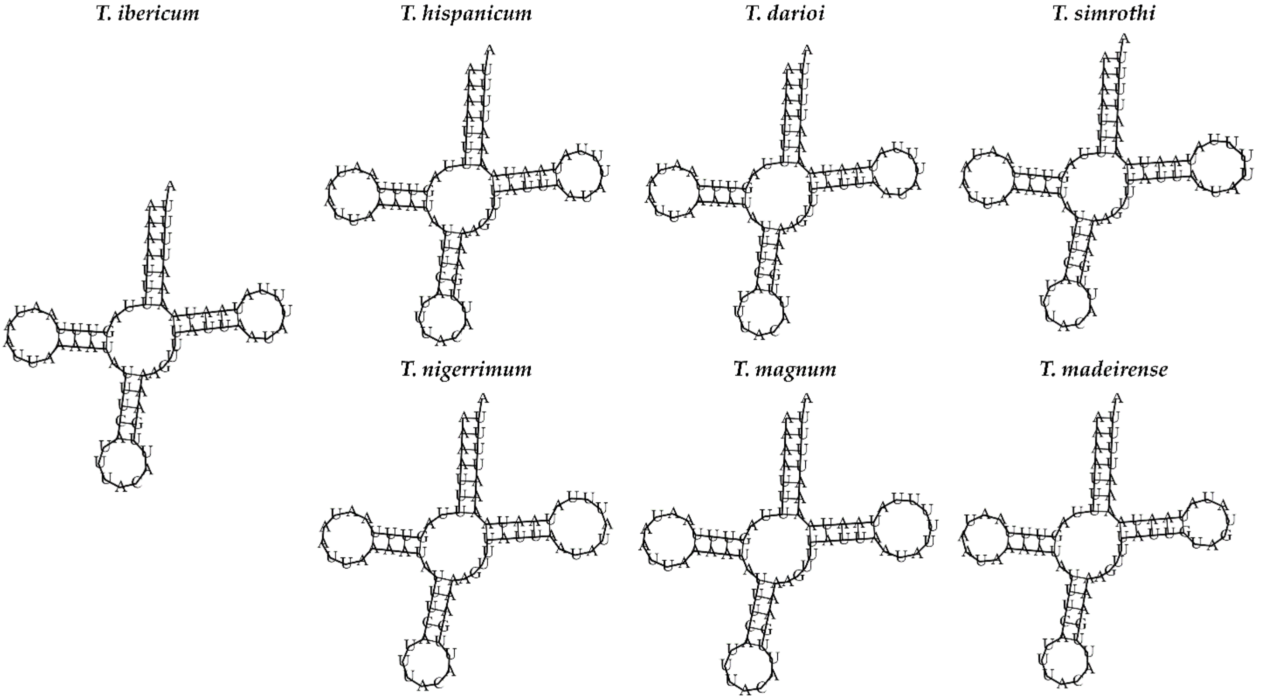

Supplement: Supplementary file 1 [file insects-15-00957-s001.zip › Supplementary Figure S1 tRNAs.pdf]
