# Supplementary material for: A Comparative Analysis of Mitogenomes in Species of the Tapinoma nigerrimum Complex and Other Species of the Genus Tapinoma (Formicidae, Dolichoderinae)
Source: Insects. 2024 Dec 2;15(12):957. doi: 10.3390/insects15120957 (PMC11677639; doi:10.3390/insects15120957)

Figure S2. Alignment of the control regions in *Tapinoma* species and potential secondary structures.

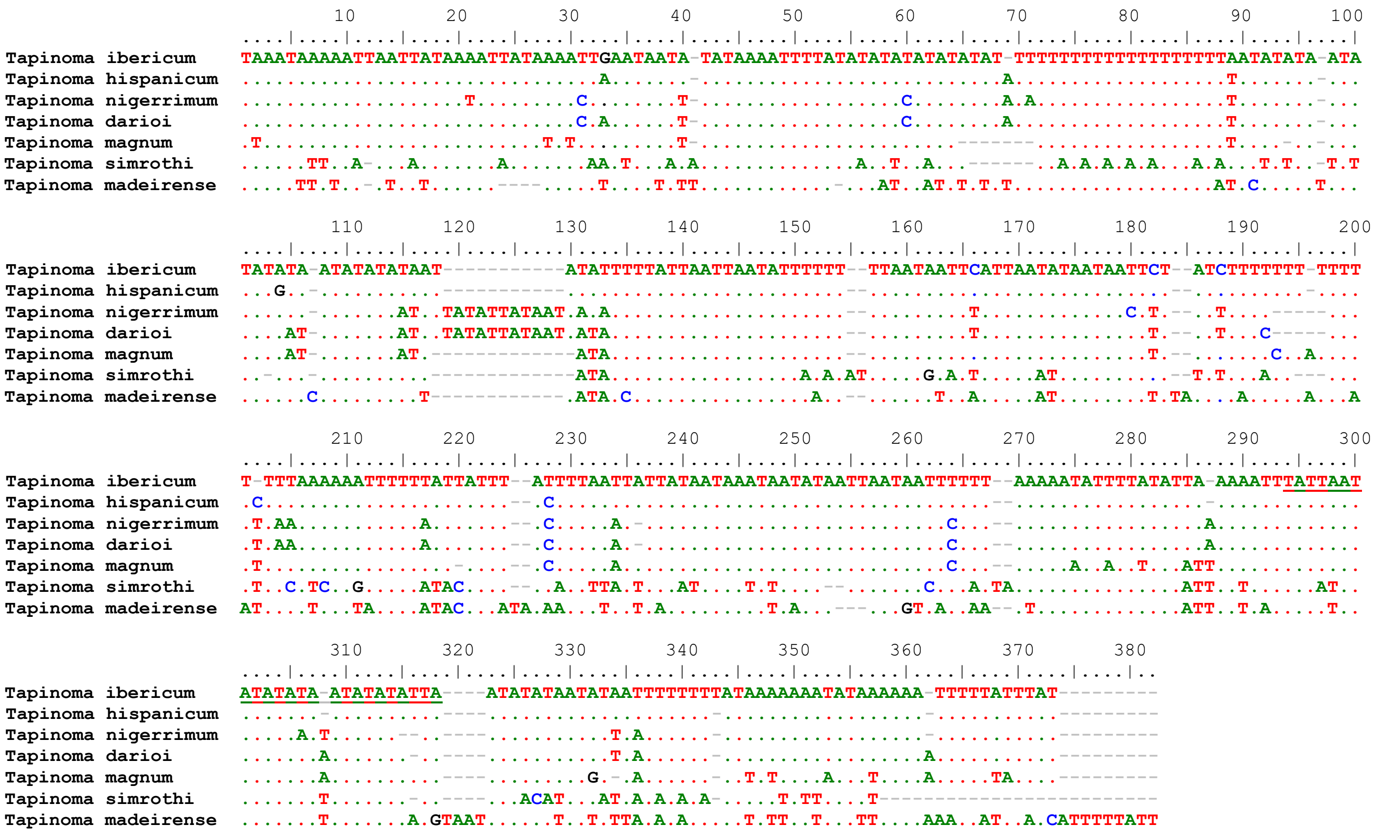

T. ibericum

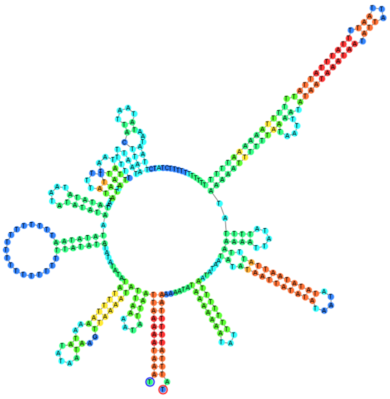

T. hispanicum

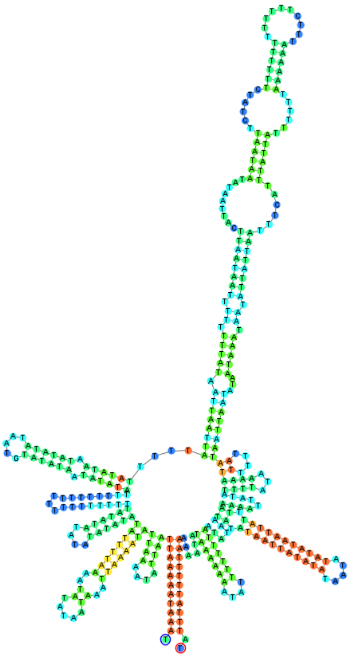

T. nigerrimum

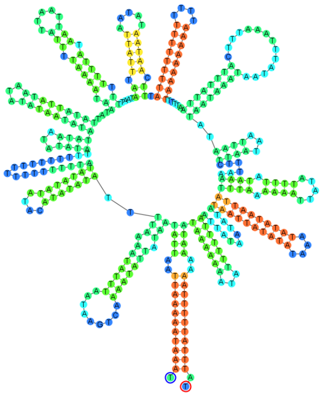

T. darioi

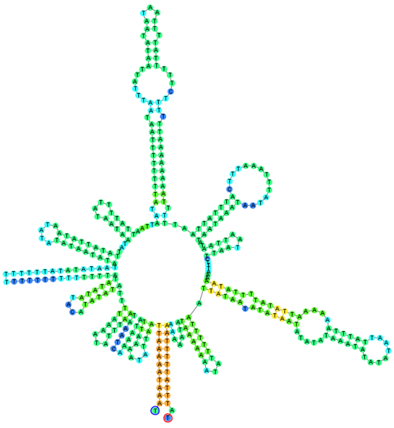

T. magnum

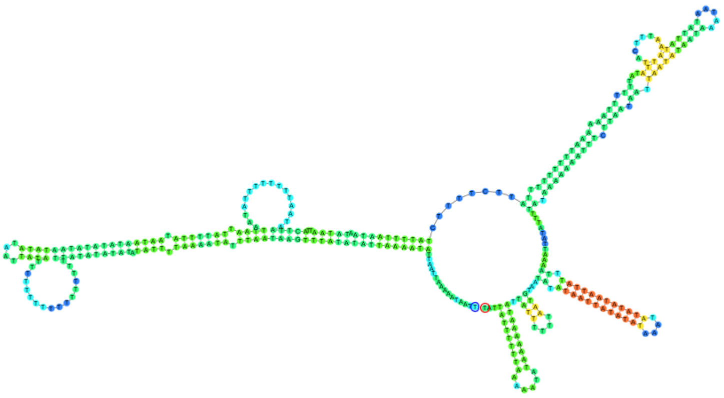

T. simrothi

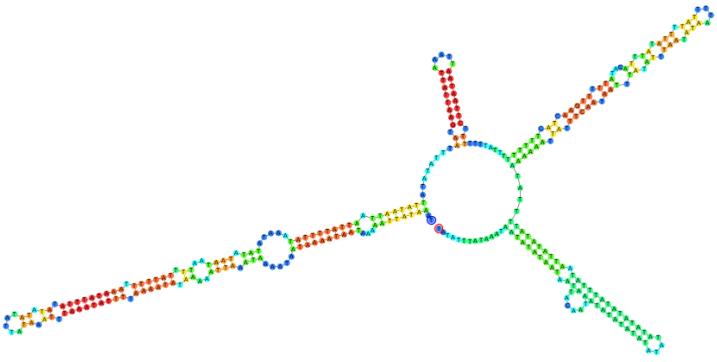

T. madeirense

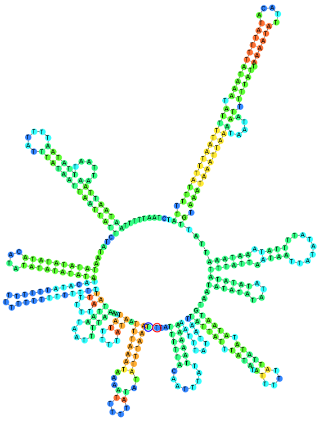

Supplement: Supplementary file 1 [file insects-15-00957-s001.zip › Supplementary Figure S2 CRs.pdf]
